# Exogenous melatonin enhances the growth and production of bioactive metabolites in *Lemna aequinoctialis* culture by modulating metabolic and lipidomic profiles

**DOI:** 10.1186/s12870-022-03941-x

**Published:** 2022-11-25

**Authors:** GahYoung Baek, Hwanhui Lee, JuHee Ko, Hyung-Kyoon Choi

**Affiliations:** College of Pharmacy, Chung-Ang University, 06974 Seoul, Republic of Korea

**Keywords:** *Lemna aequinoctialis*, Melatonin, Metabolites, Lipids, GC-MS, nanoESI-MS

## Abstract

**Background:**

*Lemna* species are cosmopolitan floating plants that have great application potential in the food/feed, pharmaceutical, phytoremediation, biofuel, and bioplastic industries. In this study, the effects of exogenous melatonin (0.1, 1, and 10 µM) on the growth and production of various bioactive metabolites and intact lipid species were investigated in *Lemna aequinoctialis* culture.

**Results:**

Melatonin treatment significantly enhanced the growth (total dry weight) of the *Lemna aequinoctialis* culture. Melatonin treatment also increased cellular production of metabolites including β-alanine, ascorbic acid, aspartic acid, citric acid, chlorophyll, glutamic acid, phytosterols, serotonin, and sucrose, and intact lipid species; digalactosyldiacylglycerols, monogalactosyldiacylglycerols, phosphatidylinositols, and sulfoquinovosyldiacylglycerols. Among those metabolites, the productivity of campesterol (1.79 mg/L) and stigmasterol (10.94 mg/L) were the highest at day 28, when 10 µM melatonin was treated at day 7.

**Conclusion:**

These results suggest that melatonin treatment could be employed for enhanced production of biomass or various bioactive metabolites and intact lipid species in large-scale *L. aequinoctialis* cultivation as a resource for food, feed, and pharmaceutical industries.

**Supplementary Information:**

The online version contains supplementary material available at 10.1186/s12870-022-03941-x.

## Background

*Lemna aequinoctialis* is a member of the aquatic monocotyledonous *Lemnaceae* family (duckweeds) and is one of the simplest and smallest plants [1, 2]. Duckweeds usually inhabit ponds, pools, and lakes across the globe, ranging from tropical to boreal regions [3, 4]. They grow rapidly (doubling time between 1.34 and 4.54 days), and have numerous potential applications in food/feed, pharmaceutical, phytoremediation, biofuel, and bioplastic industries [5–10]. Members of the *Lemnaceae* family including the *Lemna* species contain a high amount of primary metabolites, such as amino acids and starch, and a variety of secondary metabolites, such as flavonoids, hydroxycinnamic acids, and terpenoids [6, 11].

Melatonin has multiple functions in plants. As an amphiphilic molecule, melatonin is ubiquitous in all intracellular plant compartments [12], and was reported to play key roles in various plant responses and development [13]. Recently, melatonin was suggested as a plant hormone when the first phytomelatonin receptor, CAND2/PMTR1, in *Arabidopsis thaliana*, which governs stomatal closure in response to stress, was discovered [14]. It has also been reported that melatonin stimulates lateral and adventitious root formation in *Arabidopsis thaliana* through an auxin-independent mechanism [15]. Exogenous melatonin regulates the flowering and bud formation of apple trees, and similarly to animals, also acts as a signal of environmental light that regulates plant reproduction [16]. Melatonin has been proven to protect plants from reactive oxygen species (ROS) and reactive nitrogen species (RNS) by reducing free radical production [17–19]. In addition, it has been suggested that melatonin can directly scavenge reactive species through mechanisms, such as radical adduct formation, hydrogen atom transfer, and single electron transfer [20]. Melatonin has also been reported to enhance antioxidant activity by increasing the levels of the endogenous antioxidants ascorbic acid and glutathione [21]. Furthermore, it increases the efficiency of the electron transport chain by scavenging ROS and RNS produced in the mitochondria and reducing the loss of glutathione and protein damage [22]. Melatonin also protects plants from various abiotic and biotic stresses, such as acid rain, cold, drought, pathogens, infections, heavy metals, herbicides, salinity, and high temperature [23–27], and stabilizes the photosynthetic network under light and temperature stress conditions [13, 28–30].

The main aim of this study is to investigate the effect of melatonin on the growth, metabolic/lipidomic profile, and bioactive metabolite production of *L. aequinoctialis* culture and to suggest its potential application in various industries. We hypothesized that melatonin treatment would modulate the growth and production of bioactive metabolites and lipids in *L. aequinoctialis* cultures. Gas chromatography-mass spectrometry (GC-MS) and nano-electrospray ionization-mass spectrometry (nanoESI-MS) were employed to analyze various metabolites and intact lipid species in melatonin-treated *L. aequinoctialis* cultures.

## Results

### Effect of exogenous melatonin on the growth of *L. aequinoctialis*.

**Fig. 1:**
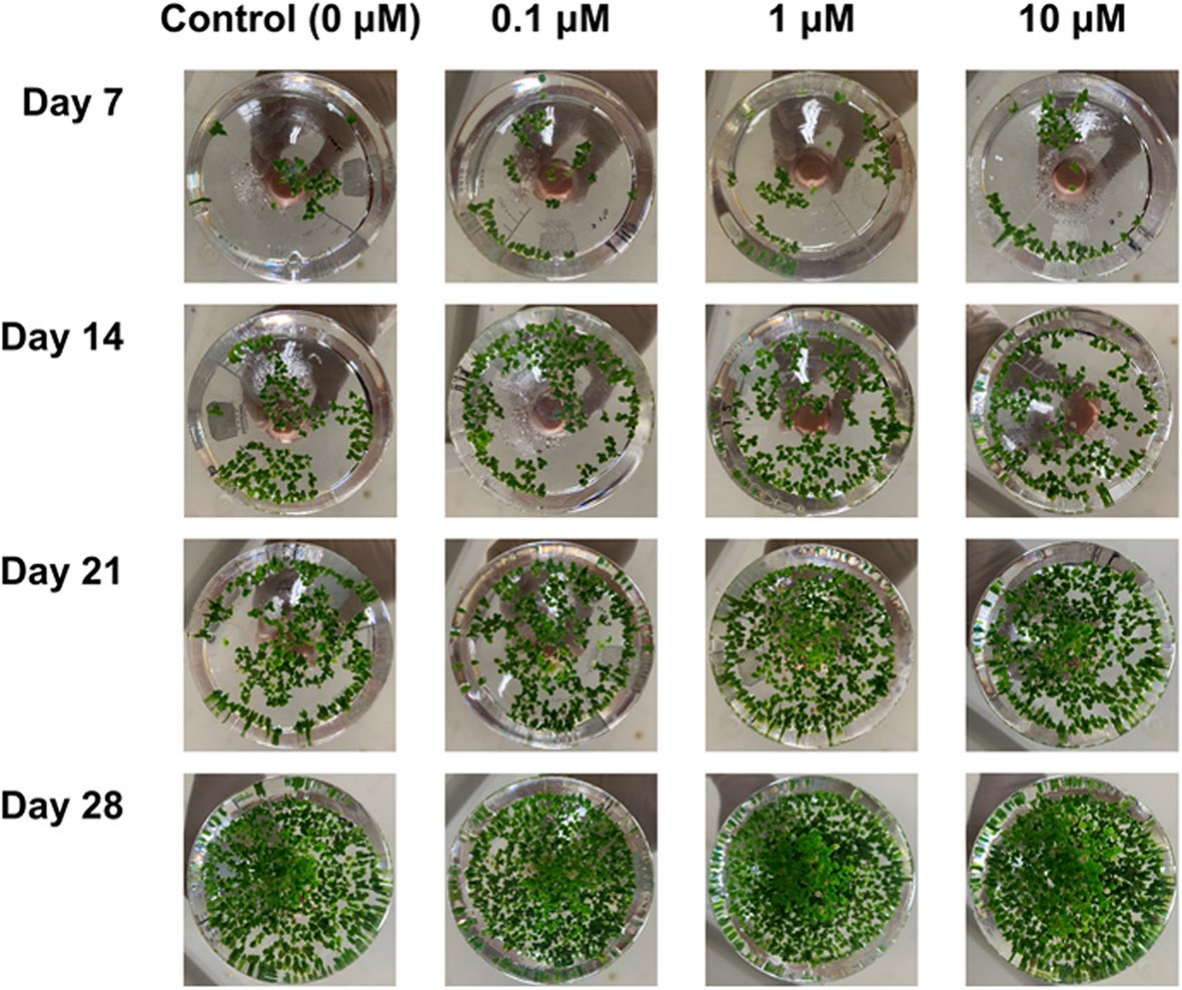
* L. aequinoctialis* culture under various melatonin treatment concentrations at days 7, 14, 21, and 28

**Fig. 2:**
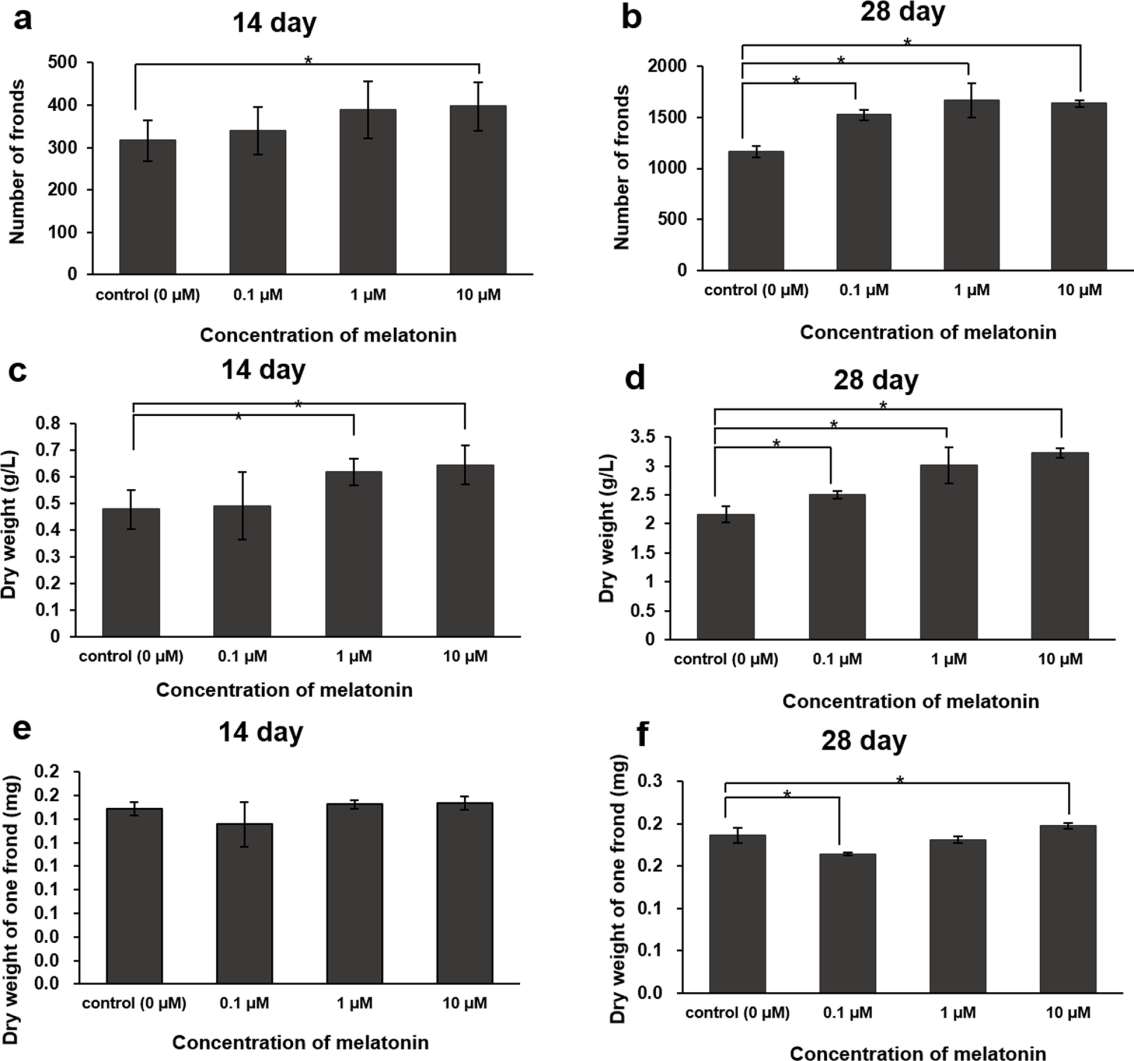
Effects of various melatonin treatment concentrations on the growth of *L. aequinoctialis* culture. Total number of fronds at day 14 (**A**) and day 28 (**B**); total dry weight at day 14 (**C**) and day 28 (**D**); and dry weight of one frond at day 14 (**E**) and day 28 (**F**). Vertical bars and error bars indicate the mean and standard deviation (*n* = 3) of each treatment group. An asterisk denotes significant differences (*p* < 0.05) between the control and treatment groups based on the Mann-Whitney test

Figure 1 shows photographs of cultivated *L*. *aequinoctialis*, which were taken every 7 days. As shown in Fig. 2, when compared to the control group, the total number of fronds harvested at day 14 was significantly higher only in the 10 µM melatonin treatment group. At day 28, it was significantly higher in the 0.1, 1, and 10 µM melatonin treatment groups. When compared to the control group, the dry weight (g/L) significantly increased in the 1 and 10 µM melatonin treatment groups at day 14, and in the 0.1, 1, and 10 µM melatonin treatment groups at day 28. In the 1 µM melatonin treatment group, there was no difference in the dry weight per frond. In addition, in the 0.1 and 10 µM melatonin treatment groups, both the dry weight per frond and the number of fronds increased, thus increasing the total dry weight.

### Effect of melatonin treatment on the metabolic profiles in *L. aequinoctialis* culture.

**Fig. 3:**
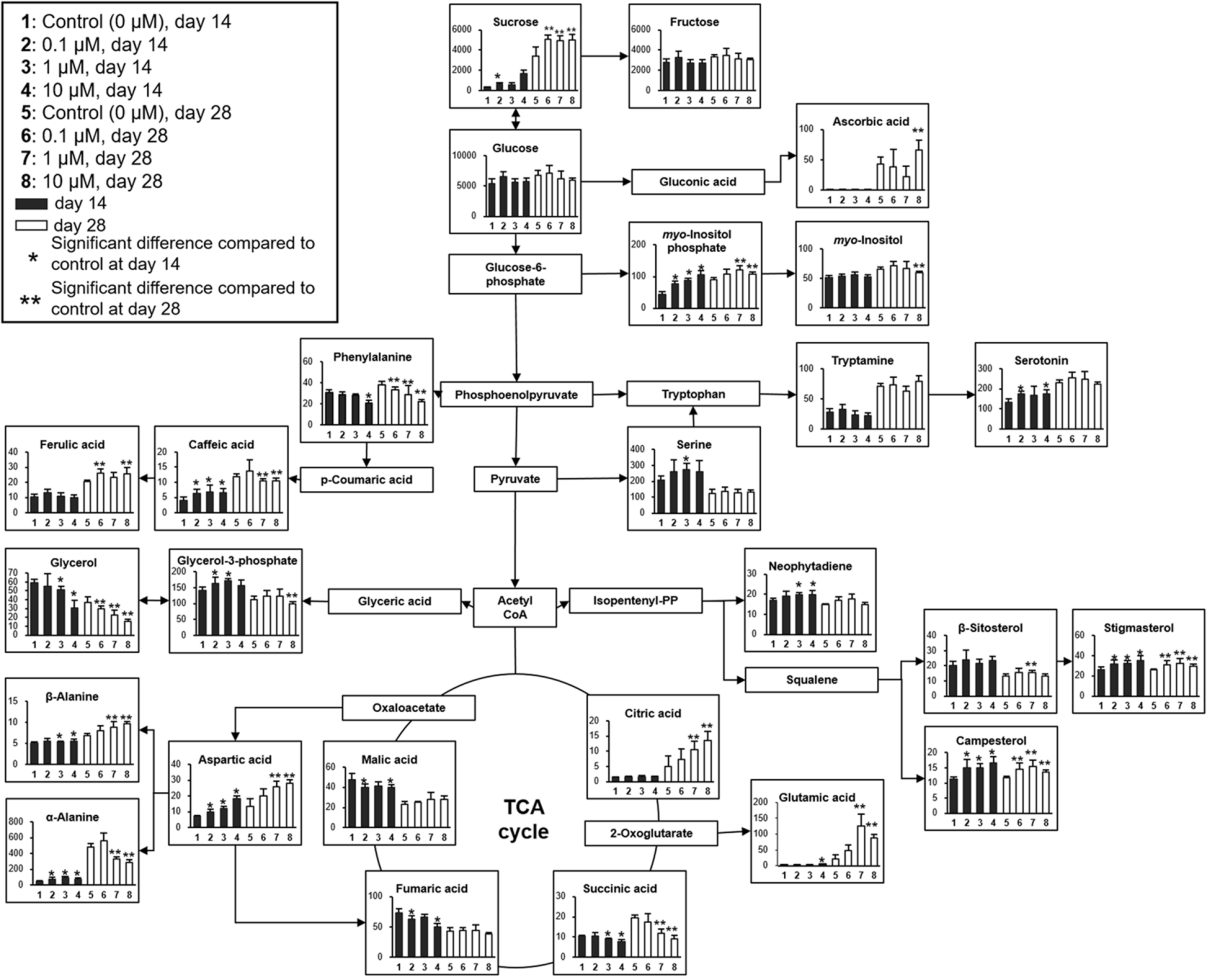
Relative levels of *L. aequinoctialis* culture metabolites under various melatonin concentrations. A pathway diagram of the relative levels (relative intensity/g) of metabolites was proposed in the Kyoto Encyclopedia of Genes and Genomes (KEGG) database (http://www.genome.jp/kegg/). Data were normalized to an internal standard (myristic-*d*_27_ acid) and then multiplied by 1,000 times for simplicity. Vertical bars and error bars indicate the mean and standard deviation (*n* = 9, three biological replicates and three technical replicates) of the control group and the 0.1, 1, and 10 µM melatonin treatment groups on days 14 and 28. Black bars indicate no significant differences, red bars indicate statistically significant increases, and blue bars indicate statistically significant decreases by using the Kruskal-Wallis test followed by the Mann-Whitney test as a post hoc analysis with Bonferroni’s correction (*p* < 0.0083)

Table S1 lists the various *L*. *aequinoctialis* metabolites detected by GC-MS analysis. A total of 49 metabolites were detected: four alcohols, 15 amino acids, four fatty acids, nine organic acids, two phenolics, three phytosterols, four sugars, and eight other assorted compounds. Figure 3 shows the relative levels (relative intensity/g) of *L*. *aequinoctialis* metabolites cultivated under 0.1, 1, and 10 µM melatonin concentrations at days 14 and 28 (Table S2 and S3, respectively). The relative levels (relative intensity/g) of α-alanine, β-alanine, aspartic acid, caffeic acid, campesterol, glycerol-3-phosphate, serine, serotonin, stigmasterol, and sucrose were significantly increased at day 14 in the melatonin treatment groups compared to the control group. The relative levels (relative intensity/g) of β-alanine, ascorbic acid, aspartic acid, campesterol, citric acid, ferulic acid, glutamic acid, β-sitosterol, stigmasterol, and sucrose were significantly increased at day 28 in the melatonin treatment groups compared to the control group. We speculate that melatonin supplementation induced the increased levels of various metabolites in *L*. *aequinoctialis* cultures.

### Effect of melatonin treatment on the lipidomic profiles in *L. aequinoctialis* culture

**Fig. 4:**
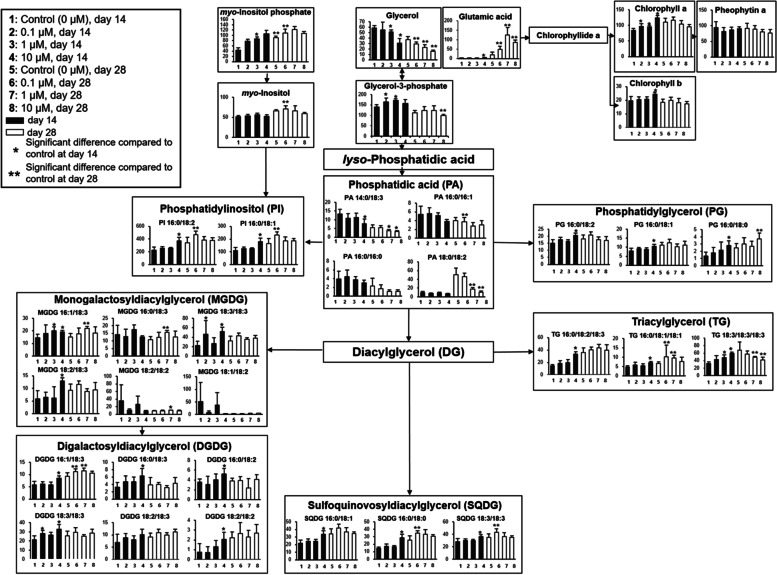
Relative levels of intact lipid species of *L. aequinoctialis* culture cultivated under various melatonin concentrations. A pathway diagram of relative (relative intensity/g) lipid levels was proposed in the Kyoto Encyclopedia of Genes and Genomes (KEGG) database (http://www.genome.jp/kegg/). The data were normalized to an internal standard (PE 17:0/17:0) and then multiplied 100 times for simplicity. Vertical bars and error bars indicate the mean and standard deviation (*n* = 9, three biological replicates and three technical replicates) of the control group and the 0.1, 1, and 10 µM melatonin treatment groups on days 14 and 28. Black bars indicate no significant differences, red bars indicate statistically significant increases, and blue bars indicate statistically significant decreases by using the Kruskal-Wallis test followed by the Mann-Whitney test as a post hoc analysis with Bonferroni’s correction (*p* < 0.0083)

Table S4 lists the various intact lipid species found in *L*. *aequinoctialis* culture detected by nanoESI-MS analysis. A total of 51 lipids were detected: six digalactosyldiacylglycerols (DGDGs), six monogalactosyldiacylglycerols (MGDGs), three phytyl derivatives, three triacylglycerols (TGs), eleven phosphatidic acids (PAs), five phosphatidylglycerols (PGs), six phosphatidylinositols (PIs), and eleven sulfoquinovosyldiacylglycerols (SQDGs). Figure 4 shows the relative lipid levels (relative intensity/g) in *L. aequinoctialis* cultivated under 0.1, 1, and 10 µM melatonin concentrations and harvested on days 14 and 28 (Table S5 and S6, respectively). When compared to the control group, the relative levels (relative intensity/g) of the DGDGs (16:1/18:3, 16:0/18:3, 16:0/18:2, 18:3/18:3, and 18:2/18:2), MGDGs (16:1/18:3, 18:3/18:3, and 18:2/18:3), phytyl derivatives (chlorophyll a and b), TGs (16:0/18:2/18:3, 16:0/18:1/18:1, and 18:3/18:3/18:3), PGs (16:0/18:2, 16:0/18:1, and 16:0/18:0), PIs (16:0/18:2 and 16:0/18:1), and SQDGs (16:0/18:1, 16:0/18:0, and 18:3/18:3) were significantly increased by day 14 in the melatonin treatment groups. When compared to the control group, the relative levels (relative intensity/g) of the DGDG (16:1/18:3), MGDGs (16:1/18:3, 16:0/18:3, and 18:1/18:2), TG (16:0/18:1/18:1), PG (16:0/18:0), PIs (16:0/18:2 and 16:0/18:1), and SQDGs (16:0/18:0 and 18:3/18:3) were significantly increased by day 28 in all melatonin treatment groups. Unlike the metabolites, which accumulated over time, the intact lipid species either increased or decreased. Nevertheless, we speculate that melatonin induced the increased levels of various intact lipid species in *L*. *aequinoctialis* cultures.

**Fig. 5:**
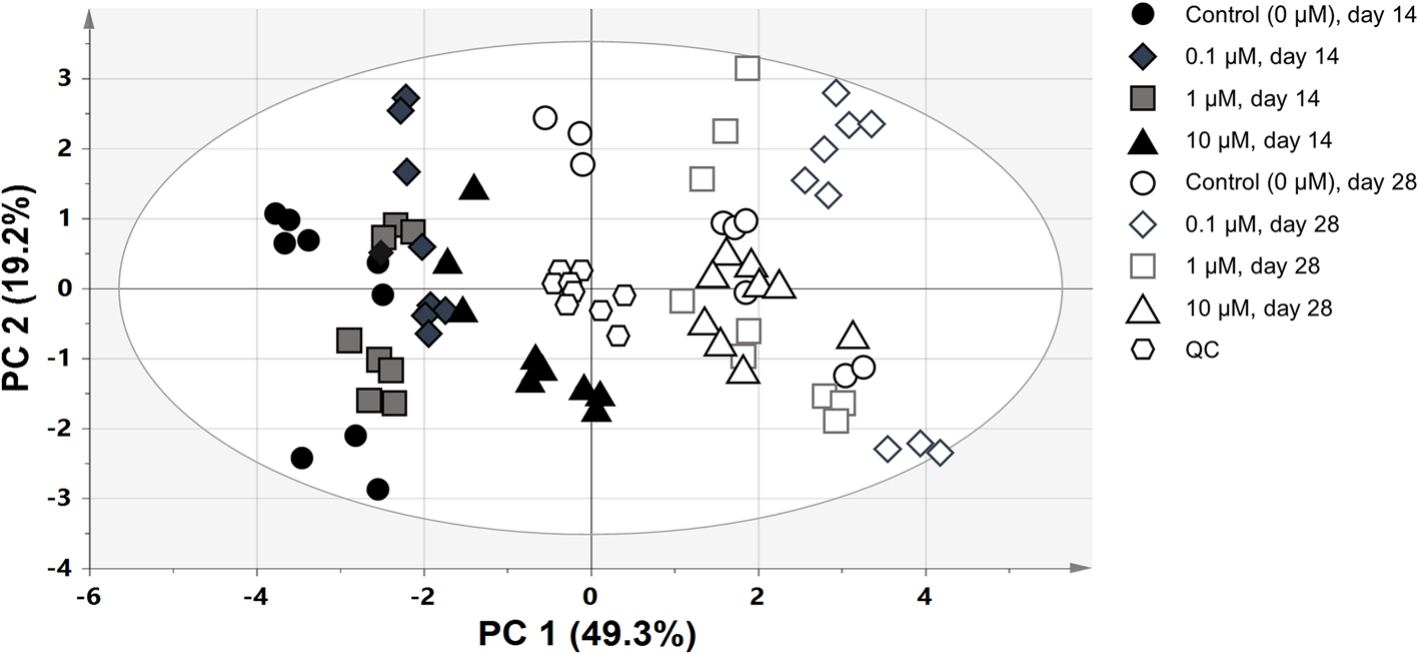
Principal component analysis (PCA)-derived score plots of metabolites and intact lipid species including QC samples of *L. aequinoctialis*. QC, quality control; control, control group (no melatonin treatment); 0.1 µM, 0.1 µM melatonin treatment; 1 µM, 1 µM melatonin treatment; 10 µM, 10 µM melatonin treatment at days 14 and 28

The metabolic and lipidomic profiles, including the quality control samples (QC samples), were plotted using PCA-derived score plots (Fig. 5). When placed in the middle of the PCA-derived score plots, the QC samples were clustered and distinctly separated from the other samples, suggesting high stability and reproducibility of GC-MS and nanoESI-MS analyses.

### Effect of various melatonin treatment time points on the productivity of *L. aequinoctialis* culture phytosterols

Exogenous melatonin treatment significantly increased the total dry weight and relative levels (relative intensity/g) of various *L. aequinoctialis* metabolites and intact lipid species and was most effective at 10 µM. To determine the productivity of *L. aequinoctialis*, a dosage of 10 µM melatonin, which increased the total frond dry weight the most, was selected based on our earlier results. This is because the relative yield (relative intensity/L) was obtained by multiplying the relative level (relative intensity/g) and total dry weight (g/L). Table 1 lists the relative yields (relative intensity/L) of the *L. aequinoctialis* culture according to various 10 µM melatonin treatment timings (days 0, 7, and 14). In the day 7 and day 14 treatment groups, the relative yields (relative intensity/L) of the comprehensive metabolites were significantly increased compared to the day 0 treatment group. The relative yields of the phytosterols were also significantly increased, reaching 142.69 relative intensity/L in the day 14 treatment group when compared to the control (81.80 relative intensity/L) and day 0 treatment groups (124.41 relative intensity/L). In this case, we speculate that melatonin acted as a growth stimulant in the early stages of the rapid growth phase of *L*. *aequinoctialis*.

**Table 1:** Relative yields of significantly altered metabolites of *L. aequinoctialis* culture cultivated under various melatonin treatment timings

No.	Compound	Control	0 Day treatment	7 Day treatment	14 Day treatment
	**Alcohols**				
1	Glycerol	33.58 ± 3.39 ^a^	51.27 ± 8.61 ^b^	43.06 ± 6.22 ^bc^	35.39 ± 7.85 ^ac^
2	Glycerol-3-phosphate	82.22 ± 32.72 ^a^	187.21 ± 13.05 ^b^	198.58 ± 11.82 ^b^	202.45 ± 9.99 ^b^
3	*myo*-Inositol	104.26 ± 7.32 ^a^	139.06 ± 7.52 ^b^	146.63 ± 17.42 ^bc^	154.66 ± 12.45 ^c^
4	*myo*-Inositol phosphate	63.31 ± 36.72 ^a^	198.98 ± 20.79 ^b^	249.53 ± 17.46 ^c^	256.15 ± 14.92 ^c^
	**Amino acids**				
5	α-Alanine	124.65 ± 20.98 ^a^	386.87 ± 158.23 ^b^	283.65 ± 93.50 ^b^	265.03 ± 20.05 ^b^
6	β-Alanine	15.98 ± 5.93 ^a^	30.51 ± 2.23 ^b^	34.28 ± 4.82 ^bc^	34.87 ± 3.13 ^c^
7	Asparagine	708.43 ± 59.66 ^a^	600.72 ± 62.76 ^b^	584.24 ± 86.45 ^b^	615.67 ± 72.98 ^ab^
8	Aspartic acid	204.76 ± 62.84 ^a^	241.07 ± 26.97 ^a^	306.24 ± 27.47 ^b^	346.88 ± 33.43 ^b^
9	Cysteine	27.35 ± 8.46 ^a^	67.80 ± 19.07 ^b^	47.99 ± 10.29 ^b^	27.91 ± 3.35 ^a^
10	Glutamic acid	410.33 ± 163.28 ^a^	737.62 ± 81.56 ^b^	1190.60 ± 138.67 ^c^	1105.45 ± 141.13 ^c^
11	Glutamine	1210.58 ± 106.67 ^a^	1366.30 ± 85.90 ^b^	1555.85 ± 97.76 ^c^	1583.48 ± 101.98 ^c^
12	Glycine	23.92 ± 4.75 ^a^	58.76 ± 15.58 ^b^	53.31 ± 10.90 ^bc^	44.62 ± 4.83 ^c^
13	Isoleucine	50.20 ± 6.79 ^a^	83.41 ± 14.77 ^b^	87.73 ± 23.49 ^b^	85.87 ± 4.54 ^b^
14	Phenylalanine	76.36 ± 9.52 ^a^	112.13 ± 8.53 ^b^	118.47 ± 27.72 ^bc^	91.43 ± 11.54 ^c^
15	Proline	72.20 ± 9.44 ^a^	98.67 ± 15.34 ^b^	86.77 ± 19.93 ^ab^	72.29 ± 5.19 ^a^
16	Pyroglutamic acid	326.03 ± 38.37 ^a^	413.59 ± 25.60 ^b^	443.75 ± 29.62 ^b^	562.92 ± 28.86 ^c^
17	Serine	198.86 ± 55.15 ^a^	392.42 ± 32.94 ^b^	398.42 ± 43.00 ^bc^	436.42 ± 22.15 ^c^
18	Threonine	126.06 ± 20.19 ^a^	196.87 ± 17.81 ^b^	211.60 ± 33.80 ^b^	206.63 ± 18.38 ^b^
19	Valine	271.53 ± 41.43 ^a^	406.61 ± 38.53 ^b^	399.42 ± 57.66 ^b^	387.16 ± 18.16 ^b^
	**Fatty acids**				
20	Linoleic acid	28.04 ± 3.91 ^a^	36.75 ± 3.13 ^b^	40.37 ± 5.98 ^bc^	42.00 ± 3.18 ^c^
21	α-Linolenic acid	38.88 ± 8.43 ^a^	56.98 ± 7.18 ^b^	63.73 ± 9.00 ^bc^	67.03 ± 4.99 ^c^
22	Palmitic acid	88.81 ± 13.69 ^a^	125.59 ± 11.07 ^b^	137.83 ± 17.68 ^bc^	147.98 ± 12.97 ^c^
23	Stearic acid	13.87 ± 2.04 ^a^	17.17 ± 2.43 ^ab^	15.95 ± 2.46 ^ab^	16.89 ± 1.55 ^b^
	**Organic acids**				
24	2-Keto-L-gluconic acid	81.94 ± 5.87 ^a^	105.42 ± 4.29 ^b^	113.85 ± 15.58 ^b^	104.13 ± 8.97 ^b^
25	3-Hydroxymethylglutaric acid	18.01 ± 2.70	16.92 ± 4.50	19.92 ± 7.86	19.62 ± 10.97
26	Citric acid	28.15 ± 5.78 ^a^	45.17 ± 2.76 ^b^	68.68 ± 27.84 ^b^	55.00 ± 7.71 ^b^
27	Erythronic acid	15.45 ± 1.95 ^a^	20.62 ± 1.46 ^b^	22.17 ± 1.94 ^b^	21.12 ± 1.13 ^b^
28	Fumaric acid	83.89 ± 6.25 ^a^	103.02 ± 5.87 ^b^	104.20 ± 11.74 ^b^	101.72 ± 7.23 ^b^
29	Glyceric acid	6.00 ± 0.60 ^a^	8.94 ± 1.70 ^b^	8.23 ± 1.70 ^b^	7.65 ± 0.90 ^b^
30	Malic acid	70.20 ± 10.57 ^a^	81.88 ± 6.79 ^ab^	87.66 ± 9.54 ^b^	91.95 ± 11.75 ^b^
31	Suberylglycine	147.28 ± 65.14 ^ab^	150.67 ± 13.52 ^a^	113.34 ± 25.98 ^b^	103.62 ± 19.48 ^b^
32	Succinic acid	20.01 ± 1.96 ^a^	35.22 ± 8.20 ^b^	33.56 ± 9.97 ^b^	30.56 ± 8.64 ^b^
	**Phenolics**				
33	Caffeic acid	29.03 ± 9.12 ^a^	43.04 ± 3.82 ^b^	50.04 ± 1.55 ^c^	46.77 ± 3.09 ^bc^
34	Ferulic acid	38.45 ± 5.93 ^a^	55.67 ± 4.43 ^b^	71.93 ± 6.58 ^c^	70.98 ± 7.12 ^c^
	**Phytosterols**				
35	Campesterol	17.89 ± 1.35 ^a^	27.89 ± 3.13 ^b^	33.88 ± 3.91 ^c^	34.03 ± 3.00 ^c^
36	β-Sitosterol	21.06 ± 2.21 ^a^	32.51 ± 3.33 ^b^	33.33 ± 2.94 ^b^	35.14 ± 1.49 ^b^
37	Stigmasterol	42.85 ± 3.80 ^a^	64.01 ± 6.02 ^b^	74.88 ± 11.80 ^b^	73.52 ± 7.03 ^b^
	**Sugars**				
38	Fructose	5586.76 ± 303.12 ^a^	7693.24 ± 706.59 ^b^	8643.45 ± 1656.10 ^b^	7639.69 ± 868.16 ^b^
39	Glucose	8542.64 ± 667.92 ^a^	14041.98 ± 1668.34 ^b^	15817.30 ± 3482.88 ^b^	14053.77 ± 2156.23 ^b^
40	Maltose	28.31 ± 4.50 ^a^	40.90 ± 7.91 ^b^	31.12 ± 5.87 ^ab^	32.16 ± 1.94 ^a^
41	Sucrose	8275.61 ± 706.61 ^a^	10322.61 ± 378.96 ^b^	12694.17 ± 1431.94 ^c^	11550.38 ± 1058.55 ^bc^
	**Others**				
42	γ-Aminobutyric acid	1432.07 ± 89.37 ^a^	2313.63 ± 415.02 ^b^	1732.92 ± 329.06 ^ac^	1823.45 ± 79.58 ^c^
43	Ascorbic acid	362.64 ± 143.07 ^a^	716.92 ± 37.00 ^b^	741.59 ± 92.36 ^b^	843.98 ± 36.24 ^c^
44	γ-Hydroxybutyric acid	2.75 ± 0.32 ^a^	3.67 ± 2.44 ^ab^	5.76 ± 3.25 ^b^	3.88 ± 0.22 ^ab^
45	Neophytadiene	19.19 ± 2.14 ^a^	29.54 ± 3.25 ^b^	35.49 ± 5.19 ^bc^	35.88 ± 3.33 ^c^
46	Phosphoric acid	4605.85 ± 652.81 ^ab^	5073.14 ± 382.64 ^a^	4356.42 ± 1260.23 ^ab^	4186.10 ± 243.49 ^b^
47	Serotonin	418.00 ± 47.04 ^a^	534.61 ± 62.80 ^b^	604.30 ± 87.23 ^b^	539.41 ± 80.05 ^b^
48	Threonic acid	56.48 ± 4.76 ^a^	56.14 ± 2.57 ^a^	59.38 ± 11.92 ^a^	40.63 ± 2.17 ^b^
49	Tryptamine	145.21 ± 26.82 ^a^	206.89 ± 7.94 ^b^	215.64 ± 24.65 ^b^	194.87 ± 13.04 ^b^

Data are mean ± standard deviation values of nine measurements from three biological replicates and three technical replicates of the control (not treated with melatonin) and various 10 µM melatonin treatment timing (Days 0,7, and 14) groups. All groups were harvested on day 28. The relative yield (relative intensity/L) was obtained by multiplying the relative levels and total dry weight (g/L). Superscript characters (a, b, and c) indicate statistically significant differences evaluated using the Kruskal-Wallis test followed by the Mann-Whitney test as a post hoc analysis with Bonferroni’s correction (*p* < 0.0083)

Among the various metabolites identified by metabolic profiling, phytosterols were selected as the bioactive metabolites for absolute quantification, and the optimal time under 10 µM melatonin treatment for the productivity of each of these metabolites was investigated. Table 2 lists the regression equation, correlation coefficient, limit of detection (LOD), and limit of quantification (LOQ) of the standard compounds (campesterol, β-sitosterol, and stigmasterol). The productivity (mg/L) of these compounds in *L. aequinoctialis* culture according to various melatonin treatment timings on days 0, 7, and 14 are listed in Table 3. The highest levels of campesterol (1.79 mg/L) and stigmasterol (10.94 mg/L) were observed in the day 7 treatment group. The highest levels of β-sitosterol (7.03 mg/L) were achieved in the day 14 treatment group.

**Table 2:** Regression equation, correlation coefficient (r^2^ values), LOD, and LOQ of phytosterol standard compounds

Compound	Regression equation	r^2^ values	LOD (µg/ml)	LOQ (µg/ml)
Campesterol	y = 0.013x – 0.0181	0.9943	0.22	0.66
β-Sitosterol	y = 0.0022x – 0.0071	0.9748	1.50	4.55
Stigmasterol	y = 0.0034x – 0.0218	0.9911	1.20	3.64

Triplicate measurements were performed for each test. LOD, limit of detection; LOQ, limit of quantification

**Table 3:** Productivity (mg/L) of phytosterols in *L. aequinoctialis* culture according to various melatonin (10 µM) treatment timings

Compounds	Control	0 Day treatment	7 Day treatment	14 Day treatment
Campesterol (mg/L)	1.15 ± 0.05 ^a^	1.53 ± 0.09 ^b^	1.79 ± 0.19 ^bc^	1.73 ± 0.13 ^c^
β-Sitosterol (mg/L)	4.11 ± 0.30 ^a^	6.08 ± 0.56 ^b^	6.80 ± 0.49 ^bc^	7.03 ± 0.39 ^c^
Stigmasterol (mg/L)	6.77 ± 0.41 ^a^	9.35 ± 0.59 ^b^	10.94 ± 1.66 ^bc^	10.88 ± 1.08 ^c^

Data are mean ± standard deviation values of nine measurements from three biological replicates and three technical replicates of the control group and the 10 µM melatonin treatment groups on days 0, 7, and 14. Superscript characters (a, b, and c) indicate statistically significant differences between the control and melatonin treatment groups by using the Kruskal-Wallis test followed by the Mann-Whitney test as a post hoc analysis with Bonferroni’s correction (*p* < 0.0083)

## Discussion

In our study, β-alanine, citric acid, and ascorbic acid levels were increased by melatonin treatment. β-alanine and citric acid are known to contribute to the antioxidant system of plants [31, 32, 30, 31]. β-alanine is also known as an osmoprotective molecule in *Gossypium herbaceum* under drought conditions [33]. Citric acid also acts as an osmolyte, assisting in the maintenance of osmotic potential and balance of sodium ions and pH in cells, thereby modulating drought and alkali stress in *Solanum tuberosum* [34, 35]. Catalase and ascorbate peroxidase, which scavenge hydrogen peroxide, are both boosted by exogenous citric acid [32]. Ascorbic acid is a stable antioxidative molecule that scavenges ROS, such as superoxide and hydroxyl radicals, either directly or through enzyme catalysis, and is involved in photosynthesis and plant defense redox pathways [36, 37]. Ascorbic acid improved the growth of *Hibiscus esculentus* by protecting amino acids and protein contents from drought-induced oxidative stress [36]. It was also reported that melatonin regulated enzymes related to the redox network, increasing the activity of ascorbate peroxidase in *Citrullus lanatus* [37]. We speculate that various antioxidative mechanisms were activated by melatonin treatment and contributed to the enhanced growth of the *L. aequinoctialis* culture.

In plants, serotonin (5-hydroxytryptamine) was involved in growth and defense processes, such as regulation of the root system, shoot organogenesis, flower development, biomass production, and defense gene expression [38]. An increased endogenous serotonin level was reported in shoots and young flower buds, suggesting that serotonin is required for shoot and flower organogenesis [39, 40]. We speculate that the melatonin-induced increase in serotonin level stimulated the growth of the *L. aequinoctialis* culture.

Plants assimilate amino acids from inorganic nitrogen sources, and nitrogen metabolism is closely related to carbon metabolism, which is essential for plant growth [41]. Nitrogen absorbed from roots is converted to aspartic acid in the presence of oxaloacetate [42]. Glutamic acid, a product of nitrogen assimilation, is synthesized via the TCA cycle and is a synthetic precursor for the synthesis of other amino acids used for chlorophyll, protein, and nucleic acid synthesis [43]. Glutamic acid and aspartic acid are also used as biostimulants, and their co-treatment increased the biomass and height of tomatoes by improving photosynthetic biochemical reactions, pore release, and CO_2_ assimilation rates [44]. Increased glutamic and aspartic acids by melatonin treatment might imply that melatonin improved nitrogen metabolism and stimulated various physiological processes in the *L. aequinoctialis* culture for enhanced growth.

Lipid metabolism is essential for plant growth, development, and environmental stress response [45]. Glycerophospholipids are major components of plant cell membranes, serving as binding sites for intracellular or intercellular proteins, providing energy for cellular metabolism, and acting as sources for the synthesis of signaling molecules related to growth, development, and survival under adverse environmental conditions [46]. Among glycerophospholipids, PI is either a membrane-derived secondary messenger that regulates signaling pathways or its precursor [47]. Increased PI levels are associated with plant cell growth, as a 5-fold higher PI level was found in elongated cells of the maize pulvini and *Arabidopsis thaliana* [48, 49]. An elevation in PI metabolism is associated with increased membrane biogenesis, cytoskeletal reorganization, and Ca^2+^ release for cell elongation by stomatal movement regulation [50]. As seen in a previous study where melatonin stimulated PI in sweet potatoes [51], melatonin treatment in our study led to increased relative levels (relative intensity/g) of PIs (16:0/18:2 and 16:0/18:1) at days 14 and 28 in the *L. aequinoctialis* cultures. This implies that melatonin greatly increased the levels of the PIs, which are major components of cell membranes and stomatal movement, and could further enhance cell proliferation in the *L. aequinoctialis* culture.

MGDG and DGDG are the two dominant galactolipids present in the thylakoid membrane and are essential for chloroplast biosynthesis and photoautotrophic growth [52]. They are important for photosynthesis and ultimately affect the development of grains and their flowers [53, 54]. SQDG is also a major lipid component of the thylakoid membrane and directly contributes by binding to the chlorophyll-protein complex to maintain the structure and function of the photosystem II complex [55, 56]. The SQDG complexes are essential for the stability of photosystem II activity under stress conditions [57]. The increased relative levels (relative intensity/g) of MGDG, DGDG, and SQDG by melatonin treatment indicate that melatonin contributed to the enhanced growth of *L. aequinoctialis* by promoting photosystem II photoreaction.

Melatonin also increased the relative levels of chlorophyll a and b in the *L. aequinoctialis* cultures at day 14 in our study. Chlorophyll is a vital molecule in plant photosynthesis, and the energy created during this process is required for carbohydrate synthesis; hence, chlorophyll conservation is important for plant survival, development, and final production [58]. Melatonin has been shown to prevent the breakdown of chlorophyll molecules [59] and upregulate ferredoxin, which protects chlorophyll from destruction by promoting the PetF gene [60]. Melatonin also boosted the photosynthetic capability in maize by increasing the chlorophyll content and upregulating the expression of photosynthetic marker genes, such as rubisco and chlorophyll A/B-binding protein [61]. Melatonin treatment resulted in increased chlorophyll levels in the *L. aequinoctialis* culture at day 14, implying that melatonin, which is associated with greater chlorophyll synthesis and slower breakdown, boosted *L. aequinoctialis* culture growth.

Phytosterols have been reported to possess low-density lipoprotein cholesterol-lowering activity in patients with type 2 diabetes [62], and antiproliferative activity in human leukemic U937 cells [63]. As listed in Table 3, the highest productivities of campesterol (1.79 mg/L) and stigmasterol (10.94 mg/L) were observed in the day 7 treatment group. The highest productivity of β-sitosterol (7.03 mg/L) was seen in the day 14 treatment group. In the day 7 and 14 treatment groups, the productivity of total phytosterols (campesterol, β-sitosterol, and stigmasterol) increased 1.63-fold, reaching 19.5 and 19.6 mg/L, respectively, compared to the control (12.0 mg/L). In addition, the productivity of total phytosterols increased 1.15 times (19.6 mg/L) in the day 14 treatment group compared to the day 0 treatment group (17.0 mg/L). The enhanced productivity of the phytosterols in the *L. aequinoctialis* cultures could be due to the increased biomass and terpenoid biosynthesis by melatonin treatment. Further studies employing transcriptomic or proteomic analyses would confirm the effect of melatonin treatment on the enhanced phytosterol biosynthesis. The higher phytosterol productivity in the day 7 and day 14 treatment groups, as compared to the day 0 treatment group, may have occurred because more *L. aequinoctialis* fronds were initially exposed to melatonin compared to those in the day 0 treatment group.

## Conclusion

In this study, we found that melatonin treatment enhanced the growth of the *L*. *aequinoctialis* culture and the production of various metabolites and intact lipid species. The importance of our study is that we obtained comprehensive metabolic and lipidomic profiles in a melatonin-treated plant system for the first time and confirmed the enhanced production of phytosterols by absolute quantification in the *L*. *aequinoctialis* culture. The increased metabolites and intact lipid species by melatonin treatment may have contributed to the increased growth of the *L. aequinoctialis* culture by enhancing several physiological functions, such as antioxidative mechanisms, nitrogen metabolism, and the photosynthetic system. Further research related to the melatonin signaling cascade, melatonin receptors, and interacting proteins using transcriptomics and proteomics could be performed to reveal more in-depth melatonin-mediated mechanisms in the future. In addition, melatonin treatment could be applied to the large-scale cultivation of *L. aequinoctialis* to produce various metabolites and intact lipid species for utilization in functional food/feed or pharmaceutical industries.

## Methods

### Cultivation of *L. aequinoctialis*

*L. aequinoctialis* (PC-10,605) was obtained from the Korean Collection for Type Cultures (KCTC; Biological Resource Center, Jeongeup, Republic of Korea), and was cultivated as previously reported [64]. Briefly, half-strength Murashige and Skoog’s basal medium supplemented with 1 mg/L benzylaminopurine, 30 g/L sucrose, and 6 g/L Gelrite were used for the cultivation of *L. aequinoctialis*, which was subcultured once every 2 weeks. The pH of the medium was adjusted to 5.8 prior to autoclaving at 121℃ for 20 min. Prior to melatonin treatment, *L. aequinoctialis* was cultivated in 1 L of liquid media for 3 days under the same environmental conditions. Then, 30 fronds of the plants were inoculated into 100 mL of liquid medium with the same composition in solid medium, except for Gelrite. The plants were cultivated in static liquid medium at 25℃, 81 ~ 84 µmol/m^2^/sec light intensity, and a 16 h light–8 h dark photoperiod in an incubator (NEX-202 M, EYELA, Nexus Technologies, Seoul, Republic of Korea).

### Melatonin treatment

*L. aequinoctialis* fronds were inoculated into 250 mL Erlenmeyer flasks (Diamond, Republic of Korea) containing 100 mL of liquid 1/2MS1BA medium and cultured at 25 °C, 6000 lx light intensity, and 16 h light–8 h dark conditions. Melatonin (0.1, 1, and 10 µM) was added to the flasks at day 0 to investigate the effect of melatonin on growth, metabolite, and intact lipid species profiles of *L. aequinoctialis* culture. The sampling timings of days 14 and 28 were determined from preliminary experiments based on the total plant growth of *L. aequinoctialis* culture (Fig. S1).

To further investigate the optimal melatonin treatment timing for enhanced productivities of selected metabolites (serotonin, campesterol, β-sitosterol, and stigmasterol), 10 µM melatonin was added to different *L. aequinoctialis* cultures on days 0, 7, and 14 (for the timed 10 µM dosage treatment). All experiments were performed in three biological replicates.

### Growth measurement

The total number of *L. aequinoctialis* fronds was counted every 7 days. To measure total dry weight, harvested whole plants were washed with distilled water and dried for 15 min. The plants were stored in a deep freezer (-70 °C) for 2 days and freeze-dried (Bondiro, Ilshin Lab. Co., Seoul, Republic of Korea) for 24 h, and the total dry weight was then measured.

### Comprehensive metabolite profiling by GC-MS analysis

Freeze-dried samples were ground with a mortar and pestle, and 20 mg of each *L. aequinoctialis* sample was separately weighed and transferred into tubes (Axygen, Union City, CA, USA). Extraction and GC-MS analysis of *L. aequinoctialis* metabolites were conducted according to the previously described method [64]. Briefly, samples were extracted with 1 mL of methanol and sonicated for 30 min. After sonication, the supernatants were filtered through 0.45 μm polytetrafluoroethylene (PTFE) syringe filters (Whatman, Maidstone, UK). Two hundred microliters of each *L. aequinoctialis* plant sample was transferred into GC vials and dried with nitrogen gas for 5 min. After derivatization, GC-MS analysis of *L. aequinoctialis* metabolites was conducted according to the previously described method [63]. Briefly, for derivatization, 30 µL of 20,000 µg/mL methoxylamine hydrochloride in pyridine, 50 µL of BSTFA (*N*,*O*-bis[trimethylsilyl]trifluoroacetamide; Sigma-Aldrich, St, Louis, MO, USA) containing 1% trimethylchlorosilane, and 10 µL of 2,000 µg/mL myristic-*d*_27_ acid (Tokyo Chemical Industry Co., Japan) in pyridine (Sigma-Aldrich, St, Louis, MO, USA) as an internal standard were added to the dried samples and the mixtures were incubated in a 65℃ water bath for 60 min. One microliter of each sample was then injected into the GC-MS (7890 A, Agilent Technologies, Santa Clara, CA, USA) with a DB-5 MS column (Agilent Technologies, Santa Clara, CA, USA), and the auxiliary, MS source, and quadrupole temperatures were set to 280, 230, and 150℃, respectively. The initial oven temperature was set to 60℃ and elevated up to 310℃ at 5℃/min.

### Comprehensive intact lipids species profiling by nanoESI-MS analysis

Five milligrams of each ground *L. aequinoctialis* sample was separately weighed, and extraction and nanoESI-MS analysis of the intact lipid species of *L. aequinoctialis* were performed as previously reported [65, 66]. Briefly, samples were added to 300 µL of methanol containing 0.01% butylated hydroxytoluene (BHT), 1,000 µL of methyl-*tert*-butyl ether (MTBE) containing 0.01% BHT, and 1,2-diheptadecanoyl-sn-glycero-3-phosphoethanolamine (PE 17:0/17:0) as an internal standard and were incubated on a shaker at room temperature for 1 h. Afterward, 250 µL of water containing 0.01% BHT was added and the samples were centrifuged, then 900 µL of the upper phase was collected. The lower phase was added to 250 µL of MTBE, 75 µL of methanol, and 62.5 µL of water, centrifuged, and then collected with the first upper phase. Next, the extracts were filtered through 0.45 μm PTFE syringe filters and 900 µL of each sample was transferred into vials and dried with nitrogen gas for 2 h. NanoESI-MS analysis of the intact lipid species of *L. aequinoctialis* was performed as previously reported [24]. The samples were analyzed in triplicate by a linear ion-trap mass spectrometer (LTQ-XL; ThermoFisher Scientific, San Jose, CA, USA) equipped with an automated nanospray ion source (TriVersa NanoMate System, Advion Biosciences, Ithaca, NY, USA). The extracts (10 µL) were infused with a nanoelectrospray chip (5.5-µm diameter spray nozzles, Advion Biosciences, Ithaca, NY, USA) and analyzed in negative and positive ion modes. The m/z range was set at 400–1200 for a 2 min run time. The full scan mode spectra method was used for analysis and tandem mass spectrometry (MS/MS) was used to identify the lipid species.

### Absolute quantification of serotonin and phytosterols

Absolute quantification of phytosterols was accomplished using the calibration curves of each standard compound. Standard curves were generated with 0.78125, 1.5625, 3.125, 6.25, 12.5, and 25 mg/L of campesterol; 0.78125, 1.5625, 3.125, 6.25, 12.5, 25, and 50 mg/L of β-sitosterol; and 3.125, 6.25, 12.5, 25, 50, and 100 mg/L of stigmasterol. GC-MS analysis for absolute quantification was performed according to the previously described method [64]. Briefly, 200 µL of each plant sample was transferred into GC vials and dried with nitrogen gas for 5 min. For derivatization, 30 µL of 20,000 µg/mL methoxylamine hydrochloride in pyridine, 50 µL of BSTFA (*N*,*O*-bis[trimethylsilyl]trifluoroacetamide; Sigma-Aldrich, St. Louis, MO, USA) containing 1% trimethylchlorosilane, and 10 µL of 2,000 µg/mL myristic-*d*_27_ acid (Tokyo Chemical Industry Co., Japan) in pyridine (Sigma-Aldrich, St. Louis, MO, USA) as an internal standard were added to the dried samples and then incubated in a 65℃ water bath for 60 min. One microliter of each sample was injected into the GC-MS (7890 A, Agilent Technologies, Santa Clara, CA, USA) with a DB-5 MS column (Agilent Technologies, Santa Clara, CA, USA), and the auxiliary, MS source, and quadrupole temperatures were set to 280, 230, and 150℃, respectively. The initial oven temperature was set to 60℃ and elevated up to 310℃ at 5℃/min. The standard calibration curves, LOD, and LOQ were also determined according to the previously reported method [64].

### Data processing and statistical analysis

SPSS Statistics 26 software (IBM, Somers, Armonk, NY., USA) was used for the Kruskal-Wallis test, Mann-Whitney test, and Bonferroni’s correction. Principal component analysis (PCA) was performed using SIMCA software (version 15.0; Umetrics, Umeå, Sweden), and mean-centered and Pareto scaling were adapted for preprocessing.

## Supplementary Information

**Additional file 1:** **Figure S1.** Growth of *L. aequinoctialis *culture cultivated for 35 days. Total number offronds (A) and dry weight (B).Dataare mean values, and the vertical bars represent the standard deviation fromthree biological replications. **TableS1.**Identification of various *L.aequinoctialis* culture metabolites by gas chromatography-mass spectrometry(GC-MS) analysis.The base peak ineach compound among ion fragments is shown in bold characters. RT, retentiontime; TMS, trimethylsilylation; MEOX, methoxylamine hydrochloride. **TableS2 **Relativelevels (relative intensity/g) of various *L.aequinoctialis* culture metabolites cultivated under various melatoninconcentrations at day 14.Data aremean ± standard deviationvalues of nine measurements from three biological replicates and threetechnical replicates of the control group and the 0.1, 1, and 10 μM melatonin treatment groups on day 14. The valueswithin a column with different letters (a, b, and c) mean statisticallysignificant differences evaluated by using the Kruskal-Wallis test followed by theMann-Whitney test as a post hoc analysis with Bonferroni's correction (*p *< 0.0083). **TableS3.**Relative levels (relative intensity/g) of various *L. aequinoctialis* culture metabolites cultivated under variousmelatonin concentrations at day 28.Dataare mean ± standard deviation values of nine measurements from three biologicalreplicates and three technical replicates of the control group and the 0.1, 1,and 10 μM melatonin treatment groups on day 28. The values within a column withdifferent letters (a, b, and c) mean statistically significant differencesevaluated by using the Kruskal-Wallis test followed by the Mann-Whitney test asa post hoc analysis with Bonferroni's correction (*p* < 0.0083). **TableS4.**Identification of various intact lipid species in *L. aequinoctialis *culture by nano-electrospray ionization-mass spectrometry(nanoESI-MS) analysis. **TableS5 **Relativelevels (relative intensity/g) of various intact lipid species of *L. aequinoctialis *culture cultivatedunder various melatonin concentrations at day 14. Data are mean ± standarddeviation values of nine measurements from three biological replicates and threetechnical replicates of the control group and the 0.1, 1, and 10 μM melatonintreatment groups on day 14. The values within a column with different letters(a, b, and c) mean statistically significant differences evaluated by using theKruskal-Wallis test followed by the Mann-Whitney test as a post hoc analysiswith Bonferroni's correction (*p* < 0.0083). **TableS6.**Relative levels (relative intensity/g) of various intact lipid species of *L. aequinoctialis* culturecultivated under various melatoninconcentrations at day 28.Data aremean ± standard deviationvalues of 9 measurements from 3 biological replicates and 3 technicalreplicates of control group and 0.1 μM, 1 μM, and 10 μMmelatonin treatment groups on day 28. The values within a column withthe different letters (a, b, and c) mean statistically significant differencesevaluated byusing the Kruskal-Wallis test followed by Mann-Whitney test as post hocanalysis with Bonferroni's correction (*p *<0.0083). 

## Data Availability

All data generated or analyzed during this study are included in this published article (and its supplementary information files).

## Abbreviations

ROS: Reactive oxygen species
RNS: Reactive nitrogen species
GC-MS: Gas chromatography-mass spectrometry
nanoESI-MS: nano-Electrospray ionization-mass spectrometry
LOD: Limit of detection
LOQ: Limit of quantification
PCA: Principal component analysis
DGDG: Digalactosyldiacylglycerol
MGDG: Monogalactosyldiacylglycerol
TG: Triacylglycerol
PA: Phosphatidic acid
PG: Phosphatidylglycerol
PI: Phosphatidylinositol
SQDG: Sulfoquinovosyldiacylglycerol
QC: Quality control
TCA: Tricarboxylic acid
