# Supplementary material for: Exogenous melatonin enhances the growth and production of bioactive metabolites in Lemna aequinoctialis culture by modulating metabolic and lipidomic profiles
Source: BMC Plant Biol. 2022 Nov 25;22:545. doi: 10.1186/s12870-022-03941-x (PMC9701026; doi:10.1186/s12870-022-03941-x)
Supplement: Supplementary file 1 — Additional file 1: Figure S1. Growth of L. aequinoctialis culture cultivated for 35 days. Total number offronds (A) and dry weight (B).Dataare mean values, and the vertical bars represent the standard deviation fromthree biological replications. TableS1.Identification of various L.aequinoctialis culture metabolites by gas chromatography-mass spectrometry(GC-MS) analysis.The base peak ineach compound among ion fragments is shown in bold characters. RT, retentiontime; TMS, trimethylsilylation; MEOX, methoxylamine hydrochloride. TableS2 Relativelevels (relative intensity/g) of various L.aequinoctialis culture metabolites cultivated under various melatoninconcentrations at day 14.Data aremean ± standard deviationvalues of nine measurements from three biological replicates and threetechnical replicates of the control group and the 0.1, 1, and 10 μM melatonin treatment groups on day 14. The valueswithin a column with different letters (a, b, and c) mean statisticallysignificant differences evaluated by using the Kruskal-Wallis test followed by theMann-Whitney test as a post hoc analysis with Bonferroni's correction (p < 0.0083). TableS3.Relative levels (relative intensity/g) of various L. aequinoctialis culture metabolites cultivated under variousmelatonin concentrations at day 28.Dataare mean ± standard deviation values of nine measurements from three biologicalreplicates and three technical replicates of the control group and the 0.1, 1,and 10 μM melatonin treatment groups on day 28. The values within a column withdifferent letters (a, b, and c) mean statistically significant differencesevaluated by using the Kruskal-Wallis test followed by the Mann-Whitney test asa post hoc analysis with Bonferroni's correction (p < 0.0083). TableS4.Identification of various intact lipid species in L. aequinoctialis culture by nano-electrospray ionization-mass spectrometry(nanoESI-MS) analysis. TableS5 Relativelevels (relative intensity/g) of various intact lipid species of L. aequinoc [file 12870_2022_3941_MOESM1_ESM.docx]

**Supporting information**

**Exogenous melatonin enhances the growth and production of bioactive metabolites in *Lemna aequinoctialis* culture by modulating metabolic and lipidomic profiles**

GahYoung Baek, Hwanhui Lee, JuHee Ko, Hyung-Kyoon Choi*

College of Pharmacy, Chung-Ang University, Seoul 06974, Republic of Korea.

***Corresponding author**

Tel: +82-2-820-5605; Fax: +82-2-816-7338; E-mail: hykychoi@cau.ac.kr

**Fig. S1 Total number of fronds (A) and dry weight (B) of *L. aequinoctialis* culture cultivated during 35 days.** Data are mean values and the vertical bars represent the standard deviation from three biological replications.


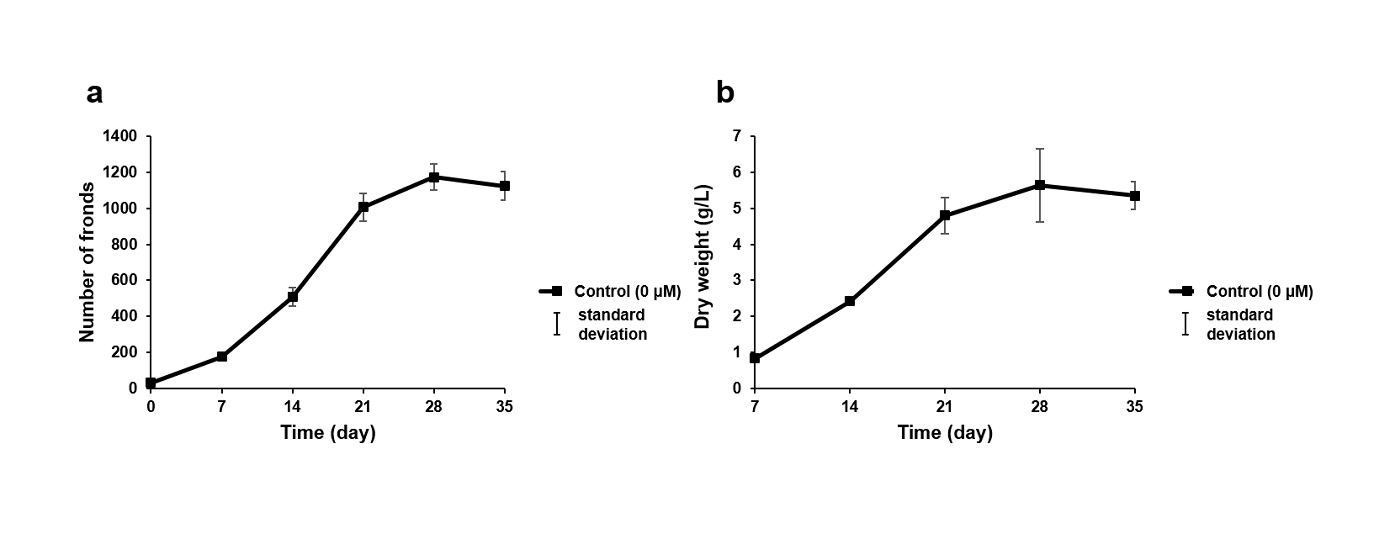


**Table S1 Identification of various metabolites in *L. aequinoctialis* culture by GC-MS analysis.**

| **No.** | **Compound** | **RT** | **Ion fragment (m/z)** | **TMS** |
| --- | --- | --- | --- | --- |
|  | **Alcohols** |  |  |  |
| 1 | Glycerol | 13.33 | 103 117 **205** 299 | 3 |
| 2 | Glycerol-3-phosphate | 25.07 | 103 299 **357** 445 | 4 |
| 3 | *myo*-Inositol | 31.46 | 191 217 **305** 318 | 6 |
| 4 | *myo*-Inositol phosphate | 36.99 | 217 299 **318** 387 | 7 |
|  | **Amino acids** |  |  |  |
| 5 | α-Alanine | 8.73 | 100 **116** 190 218 | 2 |
| 6 | β-Alanine | 17.27 | 100 **174** 248 290 | 3 |
| 7 | Asparagine | 21.38 | 100 116 130 **159** | 2 |
|  |  | 23.03 | **116** 132 188 231 | 3 |
| 8 | Aspartic acid | 19.60 | 100 218 **232** 349 | 3 |
| 9 | Cysteine | 20.39 | 100 132 **220** 294 | 3 |
| 10 | Glutamic acid | 21.95 | 128 156 **246** 348 | 3 |
| 11 | Glutamine | 25.31 | **156** 245 347 362 | 3 |
| 12 | Glycine | 14.10 | 86 **174** 248 276 | 3 |
| 13 | Isoleucine | 13.79 | **158** 218 232 260 | 2 |
| 14 | Phenylalanine | 21.99 | 192 **218** 266 294 | 2 |
| 15 | Proline | 13.87 | 133 **142** 216 244 | 2 |
| 16 | Pyroglutamic acid | 19.52 | **156** 230 258 273 | 2 |
| 17 | Serine | 12.84 | **116** 132 159 219 | 2 |
|  |  | 15.58 | 100 188 **204** 218 | 3 |
| 18 | Threonine | 16.22 | 101 117 **218** 291 | 3 |
| 19 | Valine | 11.71 | 100 **144** 218 246 | 2 |
|  | **Fatty acids** |  |  |  |
| 20 | Linoleic acid | 33.71 | 81 117 129 **337** | 1 |
| 21 | α-Linolenic acid | 33.83 | **79** 95 108 129 | 1 |
| 22 | Palmitic acid | 30.78 | **117** 129 145 313 | 1 |
| 23 | Stearic acid | 34.32 | **117** 129 273 341 | 1 |
|  | **Organic acids** |  |  |  |
| 24 | 2-Keto-L-gluconic acid | 25.44 | 103 217 **292** 421 | 5 |
| 25 | 3-Hydroxymethylglutaric acid | 21.52 | 115 231 **247** 363 | 3 |
| 26 | Citric acid | 26.27 | **273** 347 375 465 | 4 |
| 27 | Erythronic acid | 20.13 | 117 205 220 **292** | 4 |
| 28 | Fumaric acid | 15.37 | 115 133 155 **245** | 2 |
| 29 | Glyceric acid | 14.79 | 103 133 **189** 292 | 3 |
| 30 | Malic acid | 18.83 | 133 **233** 245 335 | 3 |
| 31 | Suberylglycine | 22.08 | **188** 216 231 303 | 3 |
| 32 | Succinic acid | 14.39 | 129 172 218 **247** | 2 |
|  | **Phenolics** |  |  |  |
| 33 | Caffeic acid | 32.40 | 219 307 381 **396** | 3 |
| 34 | Ferulic acid | 31.56 | 249 308 323 **338** | 2 |
|  | **Phytosterols** |  |  |  |
| 35 | Campesterol | 48.63 | **129** 343 382 472 | 1 |
| 36 | β-Sitosterol | 49.66 | **129** 357 396 486 | 1 |
| 37 | Stigmasterol | 48.93 | 55 **83** 129 484 | 1 |
|  | **Sugars** |  |  |  |
| 38 | Fructose | 25.93 | 191 **217** 257 437 | 5 |
|  |  | 26.12 |  | 5 |
|  |  | 27.35 | **103** 217 307 364 | 5(MEOX) |
|  |  | 27.55 |  | 5(MEOX) |
| 39 | Glucose | 27.76 | 191 **204** 217 435 | 5 |
|  |  | 29.52 |  | 5 |
|  |  | 27.81 | 160 205 217 **319** | 5(MEOX) |
| 40 | Maltose | 41.06 | 191 **204** 217 361 | 8 |
|  |  | 41.71 |  | 8 |
| 41 | Sucrose | 40.51 | 217 271 **361** 437 | 8 |
|  | **Others** |  |  |  |
| 42 | γ-Aminobutyric acid | 19.79 | 86 **174** 304 319 | 3 |
| 43 | Ascorbic acid | 28.79 | 205 **332** 374 449 | 4 |
| 44 | γ-Hydroxybutyric acid | 12.31 | 117 133 204 **233** | 2 |
| 45 | Neophytadiene | 26.70 | **68** 82 95 123 |  |
| 46 | Phosphoric acid | 13.24 | 133 211 **299** 314 | 3 |
| 47 | Serotonin | 37.77 | **174** 290 449 464 | 4 |
| 48 | Threonic acid | 20.55 | 205 220 **292** 319 | 4 |
| 49 | Tryptamine | 33.78 | 86 100 **174** 361 | 3 |

Base peak in each compound among ion fragments is shown as bold characters. RT, retention time; TMS, trimethylsilylation; MEOX, methoxylamine hydrochloride.

**Table S2 Relative levels (relative intensity/g) of various metabolites of *L. aequinoctialis* culture cultivated under various melatonin concentrations at day 14.**

| **No.** | **Compound** | **14day control** | **14day 0.1 μM** | **14day 1 μM** | **14day 10 μM** |
| --- | --- | --- | --- | --- | --- |
|  | **Alcohols** |  |  |  |  |
| 1 | Glycerol | 58.96 ± 4.36 ^a^ | 55.38 ± 14.34 ^ab^ | 51.57 ± 3.93 ^b^ | 30.44 ± 8.74 ^c^ |
| 2 | Glycerol-3-phosphate | 140.45 ± 11.91 ^a^ | 163.94 ± 18.41 ^b^ | 171.89 ± 7.16 ^b^ | 157.03 ± 18.38 ^ab^ |
| 3 | *myo*-Inositol | 51.66 ± 3.24 | 53.86 ± 3.75 | 56.38 ± 3.95 | 52.14 ± 4.52 |
| 4 | *myo*-Inositol phosphate | 43.15 ± 9.62 ^a^ | 77.47 ± 8.10 ^b^ | 86.99 ± 6.64 ^b^ | 105.32 ± 13.24 ^c^ |
|  | **Amino acids** |  |  |  |  |
| 5 | α-Alanine | 44.71 ± 10.83 ^a^ | 77.46 ± 22.21 ^b^ | 93.41 ± 18.86 ^b^ | 72.43 ± 21.70 ^b^ |
| 6 | β-Alanine | 4.99 ± 0.29 ^a^ | 5.59 ± 0.60 ^ab^ | 5.31 ± 0.22 ^b^ | 5.55 ± 0.42 ^b^ |
| 7 | Asparagine | 162.30 ± 62.29 ^ab^ | 150.16 ± 36.66 ^a^ | 103.13 ± 18.40 ^b^ | 66.56 ± 10.68 ^c^ |
| 8 | Aspartic acid | 7.09 ± 0.63 ^a^ | 9.79 ± 1.64 ^b^ | 12.01 ± 1.35 ^b^ | 18.28 ± 1.67 ^c^ |
| 9 | Cysteine | 1.74 ± 0.20 ^a^ | 1.86 ± 0.25 ^a^ | 1.55 ± 0.59 ^ab^ | 1.07 ± 0.11 ^b^ |
| 10 | Glutamic acid | 3.20 ± 0.72 ^a^ | 3.58 ± 0.59 ^a^ | 3.59 ± 0.60 ^a^ | 5.33 ± 0.50 ^b^ |
| 11 | Glutamine | 609.19 ± 58.59 ^ab^ | 672.45 ± 95.04 ^a^ | 576.89 ± 28.70 ^b^ | 545.42 ± 63.11 ^b^ |
| 12 | Glycine | 38.30 ± 1.69 ^a^ | 39.37 ± 5.50 ^a^ | 38.45 ± 1.38 ^a^ | 23.73 ± 3.48 ^b^ |
| 13 | Isoleucine | 29.63 ± 1.98 ^a^ | 32.28 ± 5.54 ^ab^ | 33.56 ± 1.55 ^b^ | 25.14 ± 3.51 ^c^ |
| 14 | Phenylalanine | 31.02 ± 2.56 ^a^ | 28.65 ± 2.85 ^a^ | 28.07 ± 1.56 ^a^ | 20.93 ± 2.17 ^b^ |
| 15 | Proline | 40.10 ± 2.89 ^a^ | 42.35 ± 6.92 ^a^ | 40.00 ± 1.30 ^a^ | 32.25 ± 3.42 ^b^ |
| 16 | Pyroglutamic acid | 205.72 ± 21.56 ^ab^ | 226.33 ± 18.47 ^a^ | 191.56 ± 13.32 ^b^ | 189.78 ± 13.41 ^b^ |
| 17 | Serine | 206.25 ± 25.05 ^a^ | 261.45 ± 72.61 ^ab^ | 274.11 ± 37.85 ^b^ | 260.69 ± 68.47 ^ab^ |
| 18 | Threonine | 56.27 ± 4.90 | 60.27 ± 6.05 | 59.51 ± 3.24 | 55.03 ± 5.52 |
| 19 | Valine | 137.66 ± 9.64 ^ab^ | 159.55 ± 23.95 ^a^ | 146.66 ± 7.40 ^a^ | 129.44 ± 12.98 ^b^ |
|  | **Fatty acids** |  |  |  |  |
| 20 | Linoleic acid | 40.25 ± 6.29 ^a^ | 46.84 ± 10.85 ^a^ | 44.69 ± 6.48 ^a^ | 31.13 ± 5.25 ^b^ |
| 21 | α-Linolenic acid | 89.73 ± 17.54 ^a^ | 111.63 ± 24.41 ^a^ | 102.44 ± 18.14 ^a^ | 62.02 ± 11.91 ^b^ |
| 22 | Palmitic acid | 191.80 ± 10.94 ^a^ | 193.59 ± 39.26 ^a^ | 200.37 ± 9.61 ^a^ | 131.00 ± 28.04 ^b^ |
| 23 | Stearic acid | 12.91 ± 1.32 ^a^ | 14.56 ± 2.61 ^a^ | 14.22 ± 0.64 ^a^ | 9.69 ± 0.84 ^b^ |
|  | **Organic acids** |  |  |  |  |
| 24 | 2-Keto-L-gluconic acid | 56.92 ± 4.57 | 54.10 ± 3.17 | 50.53 ± 3.85 | 51.63 ± 6.13 |
| 25 | 3-Hydroxymethylglutaric acid | 93.55 ± 15.71 ^a^ | 79.80 ± 27.85 ^ab^ | 48.78 ± 18.72 ^b^ | 20.21 ± 5.60 ^c^ |
| 26 | Citric acid | 1.43 ± 0.07 | 1.52 ± 0.31 | 1.50 ± 0.36 | 1.68 ± 0.13 |
| 27 | Erythronic acid | 9.63 ± 0.93 | 10.12 ± 2.30 | 8.66 ± 1.10 | 8.28 ± 1.71 |
| 28 | Fumaric acid | 73.14 ± 6.60^a^ | 62.47 ± 5.71 ^b^ | 65.63 ± 5.17 ^ab^ | 50.24 ± 5.33 ^c^ |
| 29 | Glyceric acid | 7.16 ± 0.37 | 7.35 ± 1.39 | 7.37 ± 1.07 | 6.02 ± 1.16 |
| 30 | Malic acid | 47.48 ± 6.15 ^a^ | 39.83 ± 3.37 ^b^ | 41.62 ± 3.55 ^ab^ | 39.99 ± 2.64 ^b^ |
| 31 | Suberylglycine | 17.93 ± 14.33 ^ab^ | 23.91 ± 8.33 ^a^ | 19.12 ± 3.87 ^a^ | 8.83 ± 5.22 ^b^ |
| 32 | Succinic acid | 10.49 ± 0.44 ^a^ | 10.35 ± 1.73 ^ab^ | 9.10 ± 0.41 ^b^ | 7.59 ± 1.11 ^c^ |
|  | **Phenolics** |  |  |  |  |
| 33 | Caffeic acid | 4.04 ± 1.07 ^a^ | 6.22 ± 1.57 ^b^ | 6.83 ± 2.20 ^b^ | 6.55 ± 1.30 ^b^ |
| 34 | Ferulic acid | 10.33 ± 1.76 | 12.92 ± 2.69 | 10.98 ± 1.98 | 9.79 ± 1.72 |
|  | **Phytosterols** |  |  |  |  |
| 35 | Campesterol | 11.24 ± 0.80 ^a^ | 14.95 ± 2.88 ^b^ | 14.93 ± 1.45 ^b^ | 16.63 ± 2.16 ^b^ |
| 36 | β-Sitosterol | 19.99 ± 2.85 | 24.00 ± 6.60 | 21.75 ± 2.68 | 23.35 ± 2.77 |
| 37 | Stigmasterol | 26.34 ± 2.35 ^a^ | 31.46 ± 4.57 ^ab^ | 32.35 ± 2.96 ^b^ | 35.44 ± 4.44 ^b^ |
|  | **Sugars** |  |  |  |  |
| 38 | Fructose | 2748.42 ± 374.61 | 3297.39 ± 564.49 | 2704.20 ± 360.33 | 2737.91 ± 329.65 |
| 39 | Glucose | 5448.62 ± 773.20 | 6565.54 ± 816.35 | 5608.52 ± 623.03 | 5790.56 ± 595.78 |
| 40 | Maltose | 54.40 ± 4.87 ^ab^ | 55.17 ± 3.48 ^a^ | 50.29 ± 2.78 ^bc^ | 45.46 ± 3.78 ^c^ |
| 41 | Sucrose | 317.33 ± 25.90 ^a^ | 657.20 ± 119.44 ^b^ | 563.54 ± 226.10 ^ab^ | 1669.04 ± 327.97 ^c^ |
|  | **Others** |  |  |  |  |
| 42 | γ-Aminobutyric acid | 1224.99 ± 74.12 ^a^ | 1287.91 ± 157.66 ^a^ | 1242.31 ± 34.85 ^a^ | 1075.82 ± 95.77 ^b^ |
| 43 | Ascorbic acid | 0.46 ± 0.05 | 0.54 ± 0.14 | 0.50 ± 0.06 | 0.48 ± 0.11 |
| 44 | γ-Hydroxybutyric acid | 1.72 ± 0.36 ^a^ | 1.62 ± 0.19 ^a^ | 1.81 ± 0.63 ^a^ | 0.98 ± 0.18 ^b^ |
| 45 | Neophytadiene | 16.96 ± 1.21 ^a^ | 18.91 ± 2.67 ^ab^ | 19.78 ± 1.00 ^b^ | 19.88 ± 2.11 ^b^ |
| 46 | Phosphoric acid | 3824.10 ± 288.25 | 3652.04 ± 240.94 | 3762.19 ± 155.71 | 3752.27 ± 295.65 |
| 47 | Serotonin | 133.83 ± 16.20 ^a^ | 174.90 ± 12.50 ^b^ | 166.96 ± 44.59 ^ab^ | 174.48 ± 19.11 ^b^ |
| 48 | Threonic acid | 13.62 ± 0.71 | 13.88 ± 3.46 | 12.61 ± 1.48 | 11.09 ± 2.70 |
| 49 | Tryptamine | 27.81 ± 6.05 ^ab^ | 32.29 ± 7.91 ^a^ | 22.88 ± 6.96 ^b^ | 22.47 ± 4.45 ^ab^ |

Data are mean ± standard deviation values of 9 measurements from 3 biological replicates and 3 technical replicates of control group and 0.1 μM, 1 μM, and 10 μM melatonin treatment groups on day 14. The values within a column with the different letters (a, b, and c) mean statistically significant differences evaluated by using the Kruskal-Wallis test followed by Mann-Whitney test as post hoc analysis with Bonferroni's correction (*p* < 0.0083).

**Table S3 Relative levels (relative intensity/g) of various metabolites of *L. aequinoctialis* culture cultivated under various melatonin concentrations at day 28.**

| **No.** | **Compound** | **28day control** | **28day 0.1 μM** | **28day 1 μM** | **28day 10 μM** |
| --- | --- | --- | --- | --- | --- |
|  | **Alcohols** |  |  |  |  |
| 1 | Glycerol | 37.14 ± 5.81 ^a^ | 29.69 ± 3.00 ^b^ | 22.58 ± 5.60 ^b^ | 15.92 ± 1.89 ^c^ |
| 2 | Glycerol-3-phosphate | 112.36 ± 10.28 ^a^ | 122.76 ± 19.03 ^ab^ | 122.85 ± 23.61 ^ab^ | 98.73 ± 6.23 ^b^ |
| 3 | *myo*-Inositol | 65.92 ± 3.08 ^a^ | 71.26 ± 7.89 ^a^ | 66.33 ± 12.87 ^ab^ | 59.53 ± 2.70 ^b^ |
| 4 | *myo*-Inositol phosphate | 90.89 ± 6.34 ^a^ | 108.52 ± 15.31 ^ab^ | 121.52 ± 12.72 ^b^ | 106.78 ± 8.49 ^b^ |
|  | **Amino acids** |  |  |  |  |
| 5 | α-Alanine | 481.32 ± 47.06 ^a^ | 560.79 ± 96.53 ^a^ | 328.17 ± 31.18 ^b^ | 290.54 ± 35.07 ^b^ |
| 6 | β-Alanine | 6.89 ± 0.52 ^a^ | 7.96 ± 1.20 ^ab^ | 8.87 ± 1.37 ^bc^ | 9.65 ± 0.52 ^c^ |
| 7 | Asparagine | 187.77 ± 4.17 ^a^ | 188.02 ± 30.89 ^ab^ | 162.85 ± 16.16 ^b^ | 127.87 ± 21.77 ^c^ |
| 8 | Aspartic acid | 13.63 ± 4.82 ^a^ | 19.96 ± 4.72 ^ab^ | 25.65 ± 3.82 ^bc^ | 27.94 ± 2.37 ^c^ |
| 9 | Cysteine | 43.14 ± 2.11 ^a^ | 37.98 ± 4.22 ^a^ | 15.84 ± 5.57 ^b^ | 7.49 ± 2.51 ^c^ |
| 10 | Glutamic acid | 20.86 ± 13.35 ^a^ | 48.92 ± 16.09 ^b^ | 124.87 ± 39.02 ^c^ | 87.15 ± 12.33 ^c^ |
| 11 | Glutamine | 393.84 ± 22.98 | 451.15 ± 74.80 | 461.04 ± 92.04 | 471.60 ± 57.69 |
| 12 | Glycine | 47.12 ± 4.51 ^a^ | 42.35 ± 6.45 ^a^ | 27.53 ± 4.20 ^b^ | 19.27 ± 4.68 ^c^ |
| 13 | Isoleucine | 40.19 ± 4.09 ^a^ | 34.97 ± 3.35 ^ab^ | 28.80 ± 5.02 ^bc^ | 28.17 ± 2.60 ^c^ |
| 14 | Phenylalanine | 38.24 ± 2.90 ^a^ | 33.27 ± 3.05 ^b^ | 29.00 ± 8.23 ^bc^ | 22.15 ± 2.21 ^c^ |
| 15 | Proline | 50.14 ± 3.03 ^a^ | 46.46 ± 4.04 ^ab^ | 36.04 ± 8.90 ^bc^ | 30.88 ± 2.16 ^c^ |
| 16 | Pyroglutamic acid | 120.77 ± 11.22 ^a^ | 139.72 ± 24.08 ^ab^ | 169.56 ± 27.97 ^b^ | 158.65 ± 23.00 ^b^ |
| 17 | Serine | 123.84 ± 27.41 | 137.92 ± 26.46 | 126.91 ± 23.90 | 131.27 ± 14.20 |
| 18 | Threonine | 64.08 ± 3.05 | 64.50 ± 10.38 | 61.24 ± 10.98 | 63.59 ± 4.00 |
| 19 | Valine | 159.56 ± 7.26 ^a^ | 160.85 ± 19.37 ^ab^ | 141.65 ± 27.56 ^ab^ | 135.81 ± 8.82 ^b^ |
|  | **Fatty acids** |  |  |  |  |
| 20 | Linoleic acid | 38.43 ± 16.16 ^a^ | 32.15 ± 7.27 ^ab^ | 26.76 ± 2.17 ^ab^ | 23.61 ± 1.97 ^b^ |
| 21 | α-Linolenic acid | 80.74 ± 44.63 ^a^ | 57.13 ± 13.69 ^ab^ | 44.16 ± 4.75 ^b^ | 39.65 ± 2.97 ^b^ |
| 22 | Palmitic acid | 108.86 ± 31.43 ^a^ | 101.42 ± 19.64 ^ab^ | 90.40 ± 8.43 ^a^ | 79.04 ± 6.23 ^b^ |
| 23 | Stearic acid | 8.25 ± 1.10 | 9.62 ± 1.98 | 9.11 ± 1.42 | 9.08 ± 1.40 |
|  | **Organic acids** |  |  |  |  |
| 24 | 2-Keto-L-gluconic acid | 53.22 ± 4.76 ^ab^ | 55.07 ± 2.38 ^a^ | 52.05 ± 10.54 ^b^ | 48.49 ± 2.38 ^ab^ |
| 25 | 3-Hydroxymethylglutaric acid | 2.58 ± 0.23 ^a^ | 2.44 ± 0.41 ^a^ | 3.53 ± 0.87 ^b^ | 5.67 ± 1.68 ^b^ |
| 26 | Citric acid | 5.03 ± 3.47 ^a^ | 7.34 ± 3.38 ^ab^ | 10.66 ± 2.68 ^b^ | 13.59 ± 2.90 ^c^ |
| 27 | Erythronic acid | 7.67 ± 0.65 ^a^ | 7.54 ± 0.93 ^ab^ | 6.99 ± 1.79 ^ab^ | 6.43 ± 0.71 ^b^ |
| 28 | Fumaric acid | 43.40 ± 5.37 | 44.27 ± 4.98 | 43.76 ± 10.04 | 38.13 ± 2.46 |
| 29 | Glyceric acid | 3.14 ± 0.36 | 2.92 ± 0.36 | 3.17 ± 0.54 | 3.14 ± 0.18 |
| 30 | Malic acid | 23.15 ± 2.72 | 25.09 ± 1.04 | 27.75 ± 7.23 | 27.83 ± 3.46 |
| 31 | Suberylglycine | 73.42 ± 11.13 ^a^ | 62.47 ± 9.73 ^a^ | 42.95 ± 8.41 ^b^ | 38.84 ± 8.02 ^b^ |
| 32 | Succinic acid | 19.46 ± 1.29 ^a^ | 17.40 ± 4.04 ^a^ | 11.80 ± 2.17 ^b^ | 9.17 ± 1.71 ^b^ |
|  | **Phenolics** |  |  |  |  |
| 33 | Caffeic acid | 11.94 ± 0.78 ^a^ | 13.75 ± 3.59 ^ab^ | 10.57 ± 0.67 ^b^ | 10.37 ± 1.01 ^b^ |
| 34 | Ferulic acid | 20.59 ± 0.90 ^a^ | 25.91 ± 2.85 ^b^ | 23.26 ± 3.39 ^ab^ | 25.86 ± 4.11 ^b^ |
|  | **Phytosterols** |  |  |  |  |
| 35 | Campesterol | 11.83 ± 0.30 ^a^ | 14.48 ± 2.05 ^b^ | 15.51 ± 2.08 ^b^ | 13.56 ± 0.87 ^b^ |
| 36 | β-Sitosterol | 13.07 ± 1.76 ^a^ | 15.40 ± 3.05 ^ab^ | 15.72 ± 1.45 ^b^ | 13.46 ± 1.33 ^a^ |
| 37 | Stigmasterol | 25.82 ± 1.27 ^a^ | 31.03 ± 4.13 ^b^ | 32.36 ± 4.71 ^b^ | 29.86 ± 1.94 ^b^ |
|  | **Sugars** |  |  |  |  |
| 38 | Fructose | 3330.71 ± 226.18 | 3489.66 ± 657.24 | 3127.32 ± 578.23 | 3037.91 ± 179.82 |
| 39 | Glucose | 6842.59 ± 722.77 | 7113.00 ± 1345.83 | 6167.21 ± 1285.30 | 5933.22 ± 390.13 |
| 40 | Maltose | 64.76 ± 2.52 ^a^ | 61.79 ± 11.18 ^ab^ | 73.26 ± 20.04 ^ab^ | 51.78 ± 3.76 ^b^ |
| 41 | Sucrose | 3411.55 ± 874.12 ^a^ | 5044.22 ± 473.62 ^b^ | 4916.30 ± 480.67 ^b^ | 5027.94 ± 561.12 ^b^ |
|  | **Others** |  |  |  |  |
| 42 | γ-Aminobutyric acid | 1335.99 ± 71.73 ^a^ | 1433.07 ± 218.89 ^a^ | 1170.97 ± 198.27 ^ab^ | 1104.72 ± 82.27 ^b^ |
| 43 | Ascorbic acid | 43.39 ± 11.55 ^a^ | 38.36 ± 28.69 ^ab^ | 22.64 ± 16.71 ^a^ | 65.90 ± 16.70 ^b^ |
| 44 | γ-Hydroxybutyric acid | 18.25 ± 1.83 ^a^ | 16.71 ± 5.67 ^a^ | 7.02 ± 0.54 ^b^ | 4.37 ± 1.48 ^c^ |
| 45 | Neophytadiene | 14.90 ± 0.45 | 16.83 ± 1.91 | 17.60 ± 2.57 | 14.95 ± 1.00 |
| 46 | Phosphoric acid | 2461.30 ± 407.14 | 2671.53 ± 343.61 | 2260.10 ± 569.29 | 2206.89 ± 265.34 |
| 47 | Serotonin | 231.21 ± 13.87 | 254.54 ± 26.17 | 247.27 ± 36.82 | 222.32 ± 11.78 |
| 48 | Threonic acid | 17.34 ± 1.88 ^a^ | 15.62 ± 1.30 ^a^ | 14.06 ± 2.70 ^ab^ | 12.41 ± 1.36 ^b^ |
| 49 | Tryptamine | 70.87 ± 4.31 ^ab^ | 73.51 ± 12.28 ^ab^ | 63.19 ± 7.14 ^a^ | 79.10 ± 8.83 ^b^ |

Data are mean ± standard deviation values of 9 measurements from 3 biological replicates and 3 technical replicates of control group and 0.1 μM, 1 μM, and 10 μM melatonin treatment groups on day 28. The values within a column with the different letters (a, b, and c) mean statistically significant differences evaluated by using the Kruskal-Wallis test followed by Mann-Whitney test as post hoc analysis with Bonferroni's correction (*p* < 0.0083).

**Table S4 Identification of various intact lipid species in *L. aequinoctialis* culture by nanoESI-MS analysis.**

| **No.** | **Lipid species** | **Ion species** | **m/z** |
| --- | --- | --- | --- |
|  | **Positive ion mode** |  |  |
|  | **Digalactosyldiacylglycerol (DGDG)** |  |  |
| 1 | DGDG 16:1/18:3 | [M + Na]^+^ | 935 |
| 2 | DGDG 16:0/18:3 | [M + Na]^+^ | 937 |
| 3 | DGDG 16:0/18:2 | [M + Na]^+^ | 939 |
| 4 | DGDG 18:3/18:3 | [M + Na]^+^ | 959 |
| 5 | DGDG 18:2/18:3 | [M + Na]^+^ | 961 |
| 6 | DGDG 18:2/18:2 | [M + Na]^+^ | 963 |
|  | **Monogalactosyldiacylglycerol (MGDG)** |  |  |
| 7 | MGDG 16:1/18:3 | [M + Na]^+^ | 773 |
| 8 | MGDG 16:0/18:3 | [M + Na]^+^ | 775 |
| 9 | MGDG 18:3/18:3 | [M + Na]^+^ | 797 |
| 10 | MGDG 18:2/18:3 | [M + Na]^+^ | 799 |
| 11 | MGDG 18:2/18:2 | [M + Na]^+^ | 801 |
| 12 | MGDG 18:1/18:2 | [M + Na]^+^ | 803 |
|  | **Phytyl Derivatives** |  |  |
| 13 | Pheophytin a | [M + H]^+^ | 871 |
| 14 | Chlorophyll a | [M + H]^+^ | 893 |
| 15 | Chlorophyll b | [M + H]^+^ | 907 |
|  | **Triacylglycerol (TG)** |  |  |
| 16 | TG 16:0/18:2/18:3 | [M + NH_4_]^+^ | 868 |
| 17 | TG 16:0/18:1/18:1 | [M + NH_4_]^+^ | 876 |
| 18 | TG 18:3/18:3/18:3 | [M + NH_4_]^+^ | 890 |
|  |  |  |  |
|  | **Negative ion mode** |  |  |
|  | **Phosphatidic acid (PA)** |  |  |
| 19 | PA 14:0/18:3 | [M - H]^-^ | 641 |
| 20 | PA 14:0/18:2 | [M - H]^-^ | 643 |
| 21 | PA 16:0/16:1 | [M - H]^-^ | 645 |
| 22 | PA 16:0/16:0 | [M - H]^-^ | 647 |
| 23 | PA 16:0/18:3 | [M - H]^-^ | 669 |
| 24 | PA 16:0/18:2 | [M - H]^-^ | 671 |
| 25 | PA 16:0/18:1 | [M - H]^-^ | 673 |
| 26 | PA 18:3/18:3 | [M - H]^-^ | 691 |
| 27 | PA 18:2/18:3 | [M - H]^-^ | 693 |
| 28 | PA 18:2/18:2 | [M - H]^-^ | 695 |
| 29 | PA 18:0/18:2 | [M - H]^-^ | 699 |
|  | **Phosphatidylglycerol (PG)** |  |  |
| 30 | PG 16:0/16:0 | [M - H]^-^ | 721 |
| 31 | PG 16:1/18:2 | [M - H]^-^ | 743 |
| 32 | PG 16:0/18:2 | [M - H]^-^ | 745 |
| 33 | PG 16:0/18:1 | [M - H]^-^ | 747 |
| 34 | PG 16:0/18:0 | [M - H]^-^ | 749 |
|  | **Phosphatidylinositol (PI)** |  |  |
| 35 | PI 16:0/18:3 | [M - H]^-^ | 831 |
| 36 | PI 16:0/18:2 | [M - H]^-^ | 833 |
| 37 | PI 16:0/18:1 | [M - H]^-^ | 835 |
| 38 | PI 18:2/18:2 | [M - H]^-^ | 857 |
| 39 | PI 18:1/18:2 | [M - H]^-^ | 859 |
| 40 | PI 18:0/18:2 | [M - H]^-^ | 861 |
|  | **Sulfoquinovosyldiacylglycerol (SQDG)** |  |  |
| 41 | SQDG 14:0/16:0 | [M - H]^-^ | 765 |
| 42 | SQDG 16:0/16:1 | [M - H]^-^ | 791 |
| 43 | SQDG 16:0/16:0 | [M - H]^-^ | 793 |
| 44 | SQDG 16:1/18:3 | [M - H]^-^ | 813 |
| 45 | SQDG 16:0/18:3 | [M - H]^-^ | 815 |
| 46 | SQDG 16:0/18:2 | [M - H]^-^ | 817 |
| 47 | SQDG 16:0/18:1 | [M - H]^-^ | 819 |
| 48 | SQDG 16:0/18:0 | [M - H]^-^ | 821 |
| 49 | SQDG 18:3/18:3 | [M - H]^-^ | 837 |
| 50 | SQDG 18:2/18:3 | [M - H]^-^ | 839 |
| 51 | SQDG 18:1/18:2 | [M - H]^-^ | 843 |

**Table S5 Relative levels (relative intensity/g) of various intact lipids species of *L. aequinoctialis* culture cultivated under various melatonin concentrations at day 14.**

| **No.** | **Lipid species** | **14day control** | **14day 0.1 μM** | **14day 1 μM** | **14day 10 μM** |
| --- | --- | --- | --- | --- | --- |
|  | **Positive ion mode** |  |  |  |  |
|  | **Digalactosyldiacylglycerol (DGDG)** |  |  |  |  |
| 1 | DGDG 16:1/18:3 | 5.79 ± 1.42 ^a^ | 6.16 ± 1.03 ^a^ | 5.90 ± 0.98 ^a^ | 8.53 ± 0.92 ^b^ |
| 2 | DGDG 16:0/18:3 | 3.28 ± 1.20 ^a^ | 4.69 ± 1.60 ^ab^ | 4.76 ± 1.22 ^ab^ | 6.30 ± 2.05 ^b^ |
| 3 | DGDG 16:0/18:2 | 3.55 ± 0.69 ^a^ | 3.12 ± 1.65 ^a^ | 4.02 ± 1.19 ^ab^ | 5.26 ± 1.05 ^b^ |
| 4 | DGDG 18:3/18:3 | 21.29 ± 4.42 ^a^ | 28.35 ± 3.13 ^b^ | 26.38 ± 3.33 ^ab^ | 33.09 ± 5.00 ^b^ |
| 5 | DGDG 18:2/18:3 | 6.88 ± 3.37 | 8.85 ± 1.68 | 7.99 ± 1.73 | 10.10 ± 2.17 |
| 6 | DGDG 18:2/18:2 | 0.77 ± 0.85 ^a^ | 0.73 ± 0.57 ^a^ | 1.32 ± 0.64 ^ab^ | 2.05 ± 0.73 ^b^ |
|  | **Monogalactosyldiacylglycerol (MGDG)** |  |  |  |  |
| 7 | MGDG 16:1/18:3 | 14.69 ± 2.75 ^a^ | 18.05 ± 6.67 ^ab^ | 20.08 ± 3.17 ^b^ | 19.06 ± 1.46 ^b^ |
| 8 | MGDG 16:0/18:3 | 14.11 ± 6.24 ^ab^ | 12.84 ± 4.93 ^ab^ | 17.40 ± 3.18^a^ | 12.76 ± 0.75 ^b^ |
| 9 | MGDG 18:3/18:3 | 21.98 ± 9.46 ^a^ | 46.46 ± 28.27 ^bc^ | 26.82 ± 11.72^ab^ | 52.11 ± 9.10 ^c^ |
| 10 | MGDG 18:2/18:3 | 5.83 ± 3.32 ^a^ | 6.49 ± 2.02 ^a^ | 6.11 ± 4.56 ^a^ | 12.94 ± 1.24 ^b^ |
| 11 | MGDG 18:2/18:2 | 35.03 ± 42.87 | 10.57 ± 3.54 | 26.07 ± 22.28 | 9.26 ± 0.54 |
| 12 | MGDG 18:1/18:2 | 51.41 ± 75.04 ^ab^ | 8.43 ± 5.76 ^ab^ | 37.44 ± 49.47 ^a^ | 3.59 ± 0.28 ^b^ |
|  | **Phytyl Derivatives** |  |  |  |  |
| 13 | Pheophytin a | 93.92 ± 19.10 | 82.11 ± 12.58 | 87.46 ± 8.35 | 91.05 ± 6.38 |
| 14 | Chlorophyll a | 83.83 ± 6.44 ^a^ | 97.18 ± 7.75 ^b^ | 95.89 ± 4.18 ^b^ | 124.41 ± 6.72 ^c^ |
| 15 | Chlorophyll b | 19.75 ± 3.05 ^a^ | 20.60 ± 1.83 ^a^ | 20.66 ± 1.86 ^a^ | 24.54 ± 1.24 ^b^ |
|  | **Triacylglycerol (TG)** |  |  |  |  |
| 16 | TG 16:0/18:2/18:3 | 14.80 ± 2.06 ^a^ | 18.26 ± 4.59 ^a^ | 19.82 ± 4.82 ^a^ | 33.53 ± 3.54 ^b^ |
| 17 | TG 16:0/18:1/18:1 | 4.82 ± 0.69 ^a^ | 5.85 ± 1.46 ^ab^ | 5.49 ± 1.05 ^a^ | 7.17 ± 0.65 ^b^ |
| 18 | TG 18:3/18:3/18:3 | 32.76 ± 3.97 ^a^ | 42.49 ± 10.95 ^abc^ | 48.35 ± 6.95 ^b^ | 59.28 ± 3.90 ^c^ |
|  |  |  |  |  |  |
|  | **Negative ion mode** |  |  |  |  |
|  | **Phosphatidic acid (PA)** |  |  |  |  |
| 19 | PA 14:0/18:3 | 13.33 ± 2.76 ^a^ | 10.97 ± 2.48 ^ab^ | 11.34 ± 2.36 ^ab^ | 7.74 ± 2.71 ^b^ |
| 20 | PA 14:0/18:2 | 7.38 ± 1.44 | 6.78 ± 1.26 | 6.84 ± 1.18 | 5.78 ± 0.94 |
| 21 | PA 16:0/16:1 | 5.40 ± 1.94 ^ab^ | 5.62 ± 1.32 ^a^ | 5.13 ± 0.65 ^a^ | 3.83 ± 0.46 ^b^ |
| 22 | PA 16:0/16:0 | 3.93 ± 1.78 | 4.48 ± 1.51 | 3.77 ± 0.67 | 3.08 ± 0.61 |
| 23 | PA 16:0/18:3 | 43.16 ± 10.86 ^ab^ | 52.24 ± 4.48 ^ab^ | 47.97 ± 3.79 ^a^ | 53.98 ± 4.63 ^b^ |
| 24 | PA 16:0/18:2 | 65.49 ± 16.09 | 76.71 ± 6.28 | 74.10 ± 6.18 | 80.45 ± 8.24 |
| 25 | PA 16:0/18:1 | 26.84 ± 6.92 | 31.00 ± 3.14 | 30.27 ± 2.70 | 32.58 ± 3.66 |
| 26 | PA 18:3/18:3 | 12.42 ± 3.23 ^ab^ | 15.24 ± 2.20 ^ab^ | 12.85 ± 0.83 ^a^ | 15.29 ± 1.24 ^b^ |
| 27 | PA 18:2/18:3 | 30.44 ± 7.93 ^ab^ | 37.43 ± 5.14 ^ab^ | 32.46 ± 2.32 ^a^ | 37.47 ± 3.50 ^b^ |
| 28 | PA 18:2/18:2 | 54.33 ± 16.98 | 64.40 ± 20.10 | 53.93 ± 6.00 | 55.39 ± 5.54 |
| 29 | PA 18:0/18:2 | 9.46 ± 3.34 | 8.11 ± 0.97 | 8.50 ± 1.74 | 7.22 ± 0.44 |
|  | **Phosphatidylglycerol (PG)** |  |  |  |  |
| 30 | PG 16:0/16:0 | 45.38 ± 7.06 | 49.82 ± 3.16 | 49.06 ± 4.51 | 56.52 ± 7.63 |
| 31 | PG 16:1/18:2 | 12.19 ± 2.03 ^ab^ | 13.11 ± 1.07 ^a^ | 13.16 ± 1.26 ^ab^ | 14.96 ± 1.30 ^b^ |
| 32 | PG 16:0/18:2 | 15.04 ± 2.42 ^a^ | 17.48 ± 1.74 ^a^ | 16.47 ± 1.04 ^a^ | 20.48 ± 1.74 ^b^ |
| 33 | PG 16:0/18:1 | 7.87 ± 1.48 ^a^ | 9.14 ± 0.67 ^a^ | 8.83 ± 0.86 ^ab^ | 10.48 ± 1.13 ^b^ |
| 34 | PG 16:0/18:0 | 1.35 ± 0.52 ^a^ | 1.78 ± 0.74 ^a^ | 2.15 ± 1.13 ^ab^ | 2.82 ± 0.63 ^b^ |
|  | **Phosphatidylinositol (PI)** |  |  |  |  |
| 35 | PI 16:0/18:3 | 48.84 ± 12.35 | 55.73 ± 7.23 | 53.10 ± 5.70 | 56.81 ± 8.43 |
| 36 | PI 16:0/18:2 | 227.52 ± 50.38 ^a^ | 258.87 ± 23.15 ^a^ | 257.92 ± 12.18 ^a^ | 369.89 ± 58.63 ^b^ |
| 37 | PI 16:0/18:1 | 109.97 ± 25.50 ^a^ | 124.54 ± 11.94 ^a^ | 124.78 ± 5.64 ^a^ | 180.68 ± 28.76 ^b^ |
| 38 | PI 18:2/18:2 | 6.00 ± 1.34 | 6.89 ± 1.30 | 5.83 ± 0.72 | 5.56 ± 0.27 |
| 39 | PI 18:1/18:2 | 3.53 ± 0.96 | 3.57 ± 1.04 | 2.72 ± 0.34 | 2.72 ± 0.23 |
| 40 | PI 18:0/18:2 | 2.73 ± 0.90 ^a^ | 3.38 ± 0.65 ^ab^ | 3.09 ± 0.81 ^ab^ | 4.27 ± 0.92 ^b^ |
|  | **Sulfoquinovosyldiacylglycerol (SQDG)** |  |  |  |  |
| 41 | SQDG 14:0/16:0 | 1.31 ± 0.67 | 1.08 ± 0.37 | 1.13 ± 0.56 | 1.35 ± 0.33 |
| 42 | SQDG 16:0/16:1 | 7.64 ± 1.62 ^ab^ | 7.80 ± 1.04 ^a^ | 7.64 ± 0.53 ^a^ | 6.60 ± 0.59 ^b^ |
| 43 | SQDG 16:0/16:0 | 28.61 ± 10.31 | 31.86 ± 8.18 | 36.85 ± 10.13 | 39.16 ± 10.42 |
| 44 | SQDG 16:1/18:3 | 25.57 ± 6.26 ^a^ | 25.84 ± 4.87 ^a^ | 25.28 ± 6.82 ^a^ | 15.27 ± 2.42 ^b^ |
| 45 | SQDG 16:0/18:3 | 122.20 ± 27.08 | 132.60 ± 13.64 | 132.28 ± 16.53 | 140.50 ± 17.68 |
| 46 | SQDG 16:0/18:2 | 58.67 ± 15.58 | 64.38 ± 9.58 | 62.16 ± 8.87 | 62.71 ± 9.79 |
| 47 | SQDG 16:0/18:1 | 21.89 ± 4.06 ^a^ | 24.36 ± 3.13 ^a^ | 24.53 ± 2.45 ^a^ | 33.60 ± 4.58 ^b^ |
| 48 | SQDG 16:0/18:0 | 14.51 ± 1.46 ^a^ | 16.95 ± 3.32 ^a^ | 16.41 ± 1.86 ^a^ | 28.52 ± 4.85 ^b^ |
| 49 | SQDG 18:3/18:3 | 27.78 ± 5.02 ^a^ | 30.16 ± 2.14 ^a^ | 29.92 ± 1.97 ^a^ | 36.11 ± 3.09 ^b^ |
| 50 | SQDG 18:2/18:3 | 29.69 ± 6.11 | 32.86 ± 3.22 | 30.87 ± 2.35 | 33.74 ± 3.11 |
| 51 | SQDG 18:1/18:2 | 10.38 ± 2.19 | 12.26 ± 4.14 | 10.61 ± 0.80 | 10.67 ± 1.26 |

Data are mean ± standard deviation values of 9 measurements from 3 biological replicates and 3 technical replicates of control group and 0.1 μM, 1 μM, and 10 μM melatonin treatment groups on day 28. The values within a column with the different letters (a, b, and c) mean statistically significant differences evaluated by using the Kruskal-Wallis test followed by Mann-Whitney test as post hoc analysis with Bonferroni's correction (*p* < 0.0083).

**Table S6 Relative levels (relative intensity/g) of various intact lipid species of *L. aequinoctialis* culture cultivated under various melatonin concentrations at day 28.**

| **No.** | **Lipid species** | **28day control** | **28day 0.1 μM** | **28day 1 μM** | **28day 10 μM** |
| --- | --- | --- | --- | --- | --- |
|  | **Positive ion mode** |  |  |  |  |
|  | **Digalactosyldiacylglycerol (DGDG)** |  |  |  |  |
| 1 | DGDG 16:1/18:3 | 9.32 ± 1.42 ^a^ | 11.29 ± 1.06 ^ab^ | 11.54 ± 1.08 ^b^ | 10.54 ± 0.80 ^ab^ |
| 2 | DGDG 16:0/18:3 | 3.94 ± 1.98 | 4.03 ± 0.47 | 3.13 ± 0.55 | 4.28 ± 1.58 |
| 3 | DGDG 16:0/18:2 | 3.79 ± 0.54 | 3.89 ± 0.81 | 2.35 ± 1.90 | 4.13 ± 0.97 |
| 4 | DGDG 18:3/18:3 | 25.91 ± 4.85 | 29.63 ± 5.13 | 25.43 ± 1.52 | 28.74 ± 4.36 |
| 5 | DGDG 18:2/18:3 | 9.15 ± 1.63 | 10.96 ± 1.42 | 9.94 ± 0.92 | 11.18 ± 1.19 |
| 6 | DGDG 18:2/18:2 | 2.23 ± 0.58 | 2.67 ± 1.11 | 2.29 ± 0.67 | 2.69 ± 0.89 |
|  | **Monogalactosyldiacylglycerol (MGDG)** |  |  |  |  |
| 7 | MGDG 16:1/18:3 | 15.06 ± 2.89 ^a^ | 17.69 ± 4.58 ^ab^ | 22.03 ± 1.42 ^b^ | 18.22 ± 4.82 ^ab^ |
| 8 | MGDG 16:0/18:3 | 10.84 ± 2.05 ^a^ | 12.48 ± 3.31 ^ab^ | 15.80 ± 1.12 ^b^ | 12.66 ± 3.87 ^ab^ |
| 9 | MGDG 18:3/18:3 | 32.39 ± 9.73 | 42.80 ± 5.52 | 35.56 ± 3.46 | 37.96 ± 6.04 |
| 10 | MGDG 18:2/18:3 | 9.19 ± 2.51 ^ab^ | 11.54 ± 1.35 ^a^ | 8.83 ± 1.02 ^b^ | 9.39 ± 2.97 ^ab^ |
| 11 | MGDG 18:2/18:2 | 8.34 ± 1.54 ^a^ | 8.97 ± 1.91 ^ab^ | 10.59 ± 0.63 ^b^ | 9.25 ± 1.96 ^ab^ |
| 12 | MGDG 18:1/18:2 | 3.48 ± 0.71 | 3.26 ± 0.96 | 3.76 ± 1.08 | 4.54 ± 1.46 |
|  | **Phytyl Derivatives** |  |  |  |  |
| 13 | Pheophytin a | 91.91 ± 16.04 | 88.88 ± 11.28 | 80.72 ± 7.33 | 76.63 ± 11.90 |
| 14 | Chlorophyll a | 110.09 ± 12.60 ^ab^ | 116.91 ± 9.94 ^a^ | 104.20 ± 11.72 ^ab^ | 95.62 ± 7.88 ^b^ |
| 15 | Chlorophyll b | 18.54 ± 2.10 | 19.95 ± 2.28 | 18.20 ± 3.18 | 17.42 ± 1.53 |
|  | **Triacylglycerol (TG)** |  |  |  |  |
| 16 | TG 16:0/18:2/18:3 | 35.69 ± 7.89 | 40.08 ± 4.89 | 43.66 ± 5.26 | 39.92 ± 8.35 |
| 17 | TG 16:0/18:1/18:1 | 6.58 ± 0.85 ^a^ | 10.16 ± 6.24 ^b^ | 9.69 ± 1.41 ^b^ | 7.71 ± 2.35 ^ab^ |
| 18 | TG 18:3/18:3/18:3 | 68.47 ± 21.59 ^a^ | 57.50 ± 7.88 ^a^ | 49.11 ± 2.17 ^b^ | 42.17 ± 7.37 ^b^ |
|  |  |  |  |  |  |
|  | **Negative ion mode** |  |  |  |  |
|  | **Phosphatidic acid (PA)** |  |  |  |  |
| 19 | PA 14:0/18:3 | 5.58 ± 1.53 ^a^ | 5.55 ± 1.20 ^a^ | 3.72 ± 0.71 ^b^ | 3.36 ± 0.48 ^b^ |
| 20 | PA 14:0/18:2 | 5.58 ± 1.54 | 5.70 ± 2.00 | 4.62 ± 0.89 | 4.94 ± 0.98 |
| 21 | PA 16:0/16:1 | 3.99 ± 0.73 ^a^ | 3.75 ± 0.98 ^ab^ | 2.78 ± 0.81 ^b^ | 3.06 ± 0.94 ^ab^ |
| 22 | PA 16:0/16:0 | 2.31 ± 1.83 | 1.73 ± 0.87 | 1.10 ± 0.39 | 1.08 ± 0.34 |
| 23 | PA 16:0/18:3 | 69.22 ± 20.00 | 78.91 ± 14.84 | 68.23 ± 8.00 | 68.08 ± 12.34 |
| 24 | PA 16:0/18:2 | 90.70 ± 25.96 | 103.40 ± 18.85 | 90.27 ± 11.48 | 88.42 ± 14.51 |
| 25 | PA 16:0/18:1 | 37.67 ± 10.03 | 42.61 ± 7.37 | 37.32 ± 4.59 | 36.21 ± 5.60 |
| 26 | PA 18:3/18:3 | 24.19 ± 6.66 ^ab^ | 27.51 ± 5.75 ^a^ | 22.21 ± 2.80 ^ab^ | 20.92 ± 3.71 ^b^ |
| 27 | PA 18:2/18:3 | 53.14 ± 14.34 | 61.23 ± 11.71 | 50.10 ± 6.69 | 47.77 ± 8.44 |
| 28 | PA 18:2/18:2 | 69.71 ± 15.25 ^ab^ | 89.16 ± 10.75 ^a^ | 75.49 ± 10.79 ^ab^ | 61.40 ± 11.96 ^b^ |
| 29 | PA 18:0/18:2 | 51.06 ± 15.04 ^a^ | 46.11 ± 8.19 ^a^ | 18.15 ± 3.07 ^b^ | 10.95 ± 2.46 ^c^ |
|  | **Phosphatidylglycerol (PG)** |  |  |  |  |
| 30 | PG 16:0/16:0 | 67.44 ± 13.13 ^ab^ | 71.91 ± 12.83 ^a^ | 54.57 ± 5.85 ^b^ | 54.13 ± 6.80 ^b^ |
| 31 | PG 16:1/18:2 | 14.39 ± 3.00 | 16.32 ± 2.28 | 13.83 ± 1.89 | 14.34 ± 2.23 |
| 32 | PG 16:0/18:2 | 18.21 ± 2.73 ^ab^ | 21.01 ± 2.21 ^a^ | 17.63 ± 1.88 ^b^ | 17.09 ± 2.73 ^ab^ |
| 33 | PG 16:0/18:1 | 11.26 ± 1.52 | 12.45 ± 1.92 | 10.27 ± 1.59 | 11.22 ± 2.03 |
| 34 | PG 16:0/18:0 | 2.49 ± 0.61 ^a^ | 3.00 ± 0.91 ^ab^ | 2.69 ± 0.69 ^a^ | 3.71 ± 0.80 ^b^ |
|  | **Phosphatidylinositol (PI)** |  |  |  |  |
| 35 | PI 16:0/18:3 | 71.48 ± 16.91 | 73.73 ± 12.54 | 60.40 ± 6.08 | 63.35 ± 11.33 |
| 36 | PI 16:0/18:2 | 344.94 ± 80.37 ^a^ | 468.95 ± 53.68 ^b^ | 383.47 ± 55.33 ^ab^ | 381.16 ± 37.86 ^a^ |
| 37 | PI 16:0/18:1 | 166.54 ± 38.82 ^a^ | 231.56 ± 24.66 ^b^ | 188.16 ± 28.05 ^ab^ | 186.77 ± 18.31 ^a^ |
| 38 | PI 18:2/18:2 | 10.79 ± 2.10 ^a^ | 11.32 ± 1.84 ^a^ | 5.82 ± 1.28 ^b^ | 4.76 ± 0.84 ^b^ |
| 39 | PI 18:1/18:2 | 5.04 ± 0.99 ^ab^ | 6.31 ± 1.35 ^a^ | 3.68 ± 0.79 ^bc^ | 3.26 ± 0.45 ^c^ |
| 40 | PI 18:0/18:2 | 3.37 ± 1.00 | 4.24 ± 1.46 | 3.46 ± 1.70 | 3.52 ± 1.13 |
|  | **Sulfoquinovosyldiacylglycerol (SQDG)** |  |  |  |  |
| 41 | SQDG 14:0/16:0 | 0.93 ± 0.33 | 1.00 ± 0.43 | 1.06 ± 0.40 | 1.27 ± 0.16 |
| 42 | SQDG 16:0/16:1 | 9.68 ± 1.60 ^ab^ | 10.80 ± 2.03 ^a^ | 8.72 ± 1.59 ^ab^ | 7.86 ± 0.98 ^b^ |
| 43 | SQDG 16:0/16:0 | 31.28 ± 8.15 | 35.70 ± 9.65 | 32.22 ± 6.06 | 30.88 ± 2.65 |
| 44 | SQDG 16:1/18:3 | 13.78 ± 1.70 ^a^ | 15.89 ± 1.79 ^a^ | 12.91 ± 2.47 ^a^ | 9.38 ± 1.54 ^b^ |
| 45 | SQDG 16:0/18:3 | 135.06 ± 22.53 | 146.20 ± 25.76 | 128.23 ± 21.05 | 128.89 ± 15.03 |
| 46 | SQDG 16:0/18:2 | 71.26 ± 13.51 | 75.46 ± 15.30 | 65.27 ± 11.44 | 66.71 ± 7.33 |
| 47 | SQDG 16:0/18:1 | 34.19 ± 6.44 ^ab^ | 41.77 ± 5.04 ^a^ | 36.54 ± 5.89 ^ab^ | 34.53 ± 1.93 ^b^ |
| 48 | SQDG 16:0/18:0 | 25.45 ± 5.77 ^a^ | 34.74 ± 3.15 ^b^ | 33.38 ± 6.40 ^ab^ | 30.76 ± 2.11 ^ab^ |
| 49 | SQDG 18:3/18:3 | 34.95 ± 5.46 ^ab^ | 43.13 ± 5.44 ^a^ | 36.31 ± 6.25 ^ab^ | 34.70 ± 2.92 ^b^ |
| 50 | SQDG 18:2/18:3 | 34.67 ± 5.05 ^ab^ | 39.69 ± 4.74 ^a^ | 30.39 ± 5.45 ^b^ | 31.72 ± 4.05 ^b^ |
| 51 | SQDG 18:1/18:2 | 12.45 ± 2.21 ^ab^ | 13.73 ± 1.60 ^a^ | 13.34 ± 2.69 ^ab^ | 10.79 ± 1.45 ^b^ |

Data are mean ± standard deviation values of 9 measurements from 3 biological replicates and 3 technical replicates of control group and 0.1 μM, 1 μM, and 10 μM melatonin treatment groups on day 28. The values within a column with the different letters (a, b, and c) mean statistically significant differences evaluated by using the Kruskal-Wallis test followed by Mann-Whitney test as post hoc analysis with Bonferroni's correction (*p* < 0.0083)
